# Programmable RNA 5-methylcytosine (m^5^C) modification of cellular RNAs by dCasRx conjugated methyltransferase and demethylase

**DOI:** 10.1093/nar/gkae110

**Published:** 2024-02-15

**Authors:** Tao Zhang, Feiyu Zhao, Jinze Li, Xiaodi Sun, Xiyun Zhang, Hejun Wang, Peng Fan, Liangxue Lai, Zhanjun Li, Tingting Sui

**Affiliations:** State Key Laboratory for Diagnosis and Treatment of Severe Zoonotic Infectious Diseases, Key Laboratory for Zoonosis Research of the Ministry of Education, Institute of Zoonosis and College of Veterinary Medicine, Jilin University, Changchun, Jilin 130000,China; State Key Laboratory for Diagnosis and Treatment of Severe Zoonotic Infectious Diseases, Key Laboratory for Zoonosis Research of the Ministry of Education, Institute of Zoonosis and College of Veterinary Medicine, Jilin University, Changchun, Jilin 130000,China; State Key Laboratory for Diagnosis and Treatment of Severe Zoonotic Infectious Diseases, Key Laboratory for Zoonosis Research of the Ministry of Education, Institute of Zoonosis and College of Veterinary Medicine, Jilin University, Changchun, Jilin 130000,China; State Key Laboratory for Diagnosis and Treatment of Severe Zoonotic Infectious Diseases, Key Laboratory for Zoonosis Research of the Ministry of Education, Institute of Zoonosis and College of Veterinary Medicine, Jilin University, Changchun, Jilin 130000,China; State Key Laboratory for Diagnosis and Treatment of Severe Zoonotic Infectious Diseases, Key Laboratory for Zoonosis Research of the Ministry of Education, Institute of Zoonosis and College of Veterinary Medicine, Jilin University, Changchun, Jilin 130000,China; State Key Laboratory for Diagnosis and Treatment of Severe Zoonotic Infectious Diseases, Key Laboratory for Zoonosis Research of the Ministry of Education, Institute of Zoonosis and College of Veterinary Medicine, Jilin University, Changchun, Jilin 130000,China; State Key Laboratory for Diagnosis and Treatment of Severe Zoonotic Infectious Diseases, Key Laboratory for Zoonosis Research of the Ministry of Education, Institute of Zoonosis and College of Veterinary Medicine, Jilin University, Changchun, Jilin 130000,China; Key Laboratory of Regenerative Biology, Guangzhou Institutes of Biomedicine and Health, Chinese Academy of Sciences, Guangzhou, Guangdong 510530, China; State Key Laboratory for Diagnosis and Treatment of Severe Zoonotic Infectious Diseases, Key Laboratory for Zoonosis Research of the Ministry of Education, Institute of Zoonosis and College of Veterinary Medicine, Jilin University, Changchun, Jilin 130000,China; State Key Laboratory for Diagnosis and Treatment of Severe Zoonotic Infectious Diseases, Key Laboratory for Zoonosis Research of the Ministry of Education, Institute of Zoonosis and College of Veterinary Medicine, Jilin University, Changchun, Jilin 130000,China

## Abstract

5-Methylcytosine (m^5^C), an abundant RNA modification, plays a crucial role in regulating RNA fate and gene expression. While recent progress has been made in understanding the biological roles of m^5^C, the inability to introduce m^5^C at specific sites within transcripts has hindered efforts to elucidate direct links between specific m^5^C and phenotypic outcomes. Here, we developed a CRISPR–Cas13d-based tool, named reengineered m^5^C modification system (termed ‘RCMS’), for targeted m^5^C methylation and demethylation in specific transcripts. The RCMS editors consist of a nuclear-localized dCasRx conjugated to either a methyltransferase, *NSUN2*/*NSUN6*, or a demethylase, the catalytic domain of mouse Tet2 (ten–eleven translocation 2), enabling the manipulation of methylation events at precise m^5^C sites. We demonstrate that the RCMS editors can direct site-specific m^5^C incorporation and demethylation. Furthermore, we confirm their effectiveness in modulating m^5^C levels within transfer RNAs and their ability to induce changes in transcript abundance and cell proliferation through m^5^C-mediated mechanisms. These findings collectively establish RCMS editors as a focused epitranscriptome engineering tool, facilitating the identification of individual m^5^C alterations and their consequential effects.

## Graphical Abstract

**Graphical Abstract:**
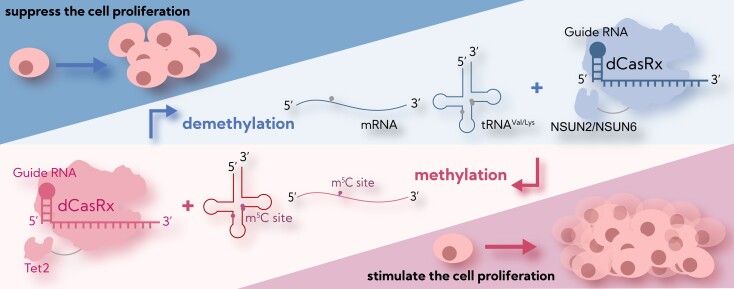

## Introduction

Post-transcriptional RNA modification is a crucial process across all organisms. With over 150 types, RNA modifications are much more abundant than DNA and protein modifications (1). Among these, the methylation of carbon 5 in cytosine (m^5^C) has gained significant attention in recent years within eukaryotes. This modification has been demonstrated to impact messenger RNA (mRNA) export (2), RNA stability (3–6), efficiency and accuracy of RNA translation (7,8), long-distance RNA transport in plants (9) and pivotal developmental processes such as nerve and brain development (10), stress response (7), gametogenesis, embryogenesis (2,11,12), mitochondrial activity (13) and diverse aspects of tumorigenesis and migration (3,14). Despite the increasing recognition of the significance of m^5^C and other RNA modifications in biology and disease, our understanding of the precise functions of m^5^C continues to lag far behind that of numerous DNA and protein modifications, even including RNA m^5^C modifications, in part because of the absence of methodologies enabling site-specific installation of RNA modifications within a target transcript within living cells.

The methylation of mRNA to form m^5^C is facilitated by members of the NOL1/NOP2/SUN domain (NSUN) protein family, consisting of seven members (NSUN1–7) in humans (15), along with the DNA methyltransferase (DNMT) homolog *DNMT2*. Recent studies have highlighted *NSUN6* as a methyltransferase exhibiting substantial substrate specificity toward mRNA, catalyzing the transfer of a methyl group from *S*-adenosyl methionine to cytosine in transfer RNA (tRNA) and within the CUCCA sequence motif in mRNA (16–18). In contrast, NSUN2, also known as NOP2/Sun domain family, member 2, demonstrates a broader substrate specificity, methylating the main parts of mRNA (2,19), the majority of expressed tRNA (2,20–22) and some noncoding RNA (23) in eukaryotes. Specifically, *NSUN2* catalyzes m^5^Cs of C48/49/50 in tRNAs (8,24,25) and m^5^C sites rich in ‘G’ across all coding sequence (CDS) regions (2).

Opposing the m^5^C ‘writer’ proteins are the ten–eleven translocation 2 (Tet2) enzymes, functioning as ‘erasers’, which have demonstrated their ability to catalyze the demethylation of m^5^C (26,27). Intriguingly, alpha-ketoglutarate-dependent dioxygenase ABH1 (ALKBH1) has been identified as a dioxygenase capable of modifying mitochondrial DNA and RNA. Specifically, it catalyzes modifications of m^5^C34 to hm^5^Cm34 (5-hydroxymethyl-2′-*O*-methylcytidine) and f5Cm34 (5-formyl-2′-*O*-methylcytidine) along mt-tRNA^Met^ and the anticodon of cytoplasmic tRNA^Leu^ (28,29). Within mRNA transcripts, m^5^C modifications recruit ‘reader’ proteins to RNA, such as Aly/REF export factor (2) and Y-box-binding protein 1 (3) (Supplementary Figure S1). Upon binding to m^5^C, these readers play diverse roles in RNA metabolism, influencing RNA export, stability, efficiency and accuracy of the translation and long-distance RNA transport in plants. Currently, the study of m^5^C in biological processes relies on global knockdown or overexpression of these m^5^C writers, erasers and readers within cells. Despite the rapid progress made in understanding the abundance, dynamics, biogenesis and localization of m^5^C, establishing methods for precisely manipulating m^5^C modifications in specific transcripts remains an ongoing challenge.

The CRISPR/Cas system has proven effective in studying the endogenous functions and dynamic variations of nucleic acids in previous studies (30–32). Cas13, a CRISPR-associated nuclease, which is a class 2 type VI CRISPR–Cas endonuclease, can bind and cleave single-stranded RNA guided by a complementary guide RNA (gRNA). Currently, several distinct Cas13 family nuclease subtypes have been identified, named as Cas13a–d, Cas13X and Cas13Y (33–39). Cas13 endonucleases exhibit high efficiency in RNA knockdown. Subsequently, the CRISPR–Cas13 system has been repurposed as a programmable tool for RNA editing (40,41), nucleic acid detection (42) and RNA epigenetic manipulation (43–45). Previous studies have demonstrated that fusing the catalytically inactive dCas13 with m^6^A demethylase ALKBH5, m^1^A demethylase ALKBH3 (46) or a truncated methyltransferase domain of methyltransferase-like 3 (44) can facilitate potent m^6^A demethylation or installation, respectively. However, efficient approaches for manipulating the m^5^C modification within specific RNAs remain yet to be established. We speculated that tethering catalytically inactive CasRx (dCasRx) to m^5^C writers or erasers could enable the programmable installation of m^5^C at sites specified by a CasRx gRNA.

In this study, we fused components of the m^5^C methyltransferase (*NSUN2*/*NSUN6*) or demethylase [Tet2 catalytic domain (CD)] to dCasRx. This successful fusion approach impacts the abundance of m^5^C at specific RNA sites and influences RNA levels. Consequently, this modified m^5^C system will aid in elucidating the causal relationships between m^5^C and biological phenotype by enabling programmable methylation of specific cytosines within RNA.

## Materials and methods

### Plasmid construction

CasRx and inactive dCasRx were acquired from Addgene (#109049, #109050). The RCMS (reengineered m^5^C modification system) editors were constructed by fusing candidate methyltransferases or demethylase to the C-terminus of dCasRx using a short linker (GSGGGGS), forming dCasRx-NSUN6 editors, dCasRx-NSUN2 editors and dCasRx-Tet2 CD editors. NSUN6 editors utilized the NSUN6 methyltransferase and NSUN2 editors utilized the NSUN2 methyltransferase, while Tet2 editors used the Tet2 demethylase catalytic domain. NSUN6 was amplified from the complementary DNA (cDNA) of HEK293T cells via polymerase chain reaction (PCR) and NSUN2 was commercially synthesized by GenScript Biotech (China) and then cloned into the dCasRx backbone. Tet2 CD was amplified from the pcDNA3-Flag-Tet2 CD plasmid (#72219), purchased from Addgene. All RCMS editors were designed for constitutive expression under an EF1α promoter. For CasRx gRNAs, the pXR003 plasmid: A CasRx gRNA cloning backbone (#109053) was used for construction, allowing constitutive gRNA expression under a U6 promoter.

The construction of dCasRx-NSUN6 and gRNAs of *RPSA* lentivirus plasmids involved PCR amplification and fusion of dCasRx-NSUN6 to the pCDH-CMV-MCS-EF1a-copGFP-T2A-puro vector backbone. Q5 polymerase (NEB) was used for the amplification process. The primers used in this work are mentioned in Supplementary Table S1.

### gRNA design

We identified cytosine sites reported as accessible to m^5^C methylation from databases GSE125046 (16) and GSE148764 (17). To ensure specificity, the designed gRNAs were assessed using NCBI BLAST (https://blast.ncbi.nlm.nih.gov/Blast.cgi) to prevent mRNA off-target binding within the human genome. A comprehensive list of the CasRx gRNAs used in this work can be found in Supplementary Table S3.

### Cell culture and transfection

Mammalian cell experiments were performed using HEK293T (ATCC) and HepG2 cell lines (ATCC). These cells were cultured in Dulbecco’s modified Eagle medium (DMEM) supplemented with GlutaMAX (Meilunbio, China), along with 10% fetal bovine serum (CLARK, FB15015) and incubated at 37°C with 5% CO_2_. The stable NSUN2^−/−^ knockout HEK293 cell lines were cultured in DMEM containing 10% fetal bovine serum.

### HEK293T transfection and lentivirus infection

HEK293T cells were seeded onto six-well poly-d-lysine plates (Corning) at a density of 5 × 10^5^ cells per well in the culture medium. Upon reaching 80% confluency, around 12 h after plating, cells were transfected with 2 μg RCMS editor plasmids and 1 μg CasRx gRNA plasmid using 8 μl Hieff Trans™ Liposomal Transfection Reagent (YEASEN, China), following the manufacturer’s instructions. The cells were then cultured for 48 h post-transfection before total RNA extraction.

For lentivirus infection, procedures followed a previous study (47). The target plasmid and packaging vectors were co-transfected into HEK293T cells. The resulting cell supernatant, rich in lentivirus particles, was concentrated via ultracentrifugation and subsequently used for virus transduction. Lentiviral vectors expressing dCasRx-NSUN6-EGFP, dCasRx-NSUN2-EGFP and dCasRx-Tet2CD-EGFP or gRNAs for dCasRx were employed in constructing stable cell lines, as previously described. Following 72 h, cells were subjected to puromycin selection (2 μg/ml) for 2–4 days.

### RNA extraction, real-time quantitative PCR

HEK293T cells were first washed with phosphate-buffered saline (PBS) and then lysed using TRIzol (TIANGEN Biotech, China). Total RNA extraction was carried out from the cells, followed by quantitative reverse transcription PCR (qRT-PCR) analysis performed according to a previously established study (48). Total RNA was extracted by adding 1000 μl TRIzol (TIANGEN Biotech, China) and 200 μl chloroform to the cells. After centrifuging at 12 000 rpm for 15 min at 4°C, the supernatant was transferred to a 1.5 ml RNase-free tube. Then, 100% isopropanol and 75% alcohol were added to precipitate and purify the RNA. All the reagents were precooled. Subsequently, cDNA was synthesized using the FastKing RT Kit (with gDNase) (TIANGEN Biotech, China) as per the manufacturer’s instructions. For qPCR assays, QuantStudio3 (Thermo Fisher Scientific) was used, employing SuperReal PreMix Plus (SYBR Green) (TIANGEN Biotech, China) with the cDNA obtained from each sample. The expression levels of the treated samples were normalized to the controls, using GAPDH or 18S as the endogenous control, and calculated using the 2^−ΔΔCT^ formula. Details regarding the primers used in this study can be found in Supplementary Table S2 (Sangon Biotech, China).

### Western blotting

Western blotting followed a previously described protocol (49). Briefly, whole-cell extracts were prepared in RIPA buffer containing phenylmethanesulfonyl fluoride (Beyotime Biotechnology) on ice for 30 min, with brief vertexing every 10 min. Subsequently, these extracts were subjected to western blotting analyses using the anti-HA antibody (Sigma, H3663), anti-GAPDH polyclonal antibody (Proteintech, 10494-1-AP), anti-RPSA antibody (Proteintech, 14533-1-AP), anti-KAT7 antibody (Proteintech, 13751-1-AP), anti-BBS4 antibody (Proteintech, 12766-1-AP), anti-TRAF7 antibody (Cusabio, CSB-PA780056) and anti-tublin antibody (Proteintech, 11224-1-AP). The images were captured using the Tanon 5200 Imaging System (Tanon).

### Bisulfite treatment and m^5^C modification detection with RT-PCR

For bisulfite treatment, total RNA was treated with the EZ RNA Methylation™ Kit (Zymo Research, USA) following the manufacturer’s protocol. The treated RNA was then used for cDNA synthesis using the above-mentioned method. Subsequently, the cDNA synthesized using the FastKing RT Kit was amplified using 2× Taq PCR Mix (TIANGEN Biotech, China) and 2× EpiArt HS Taq Master Mix (Dye Plus) (Vazyme, China). A part of the sites was subjected to analysis by either Sanger sequencing or deep sequencing, detected using HiTOM analysis (50). Another subset of sites was analyzed by transforming target RNA fragments using a PGM-T connection kit (Sangon Biotech, China) following m^5^C site-specific primer extension.

### Flow cytometry

The cells underwent digestion using 0.25% trypsin–EDTA (Meilunbio, China) and were suspended in ice-cold PBS supplemented with 10% fetal calf serum. Subsequently, the cells were analyzed using flow cytometry on BD FACSCelesta. FlowJo was used to analyze the flow cytometry data.

### mRNA BS-seq

mRNA bisulfite sequencing (BS-seq) library construction followed our previously described method (51). Total RNA was extracted from HEK293T cells, and poly(A)+ RNA was isolated from the total RNA using Oligo dT Magnetic Beads. Subsequently, the isolated RNA underwent bisulfite salt treatment. The converted RNA was fragmented and used for library construction employing E-gene. Sequencing of the libraries was performed using HiSeq X Ten (Illumina) to generate paired-end 150-bp reads.

### Immunofluorescence microscopy

Immunofluorescence, as detailed in a previous study (52), involved cloning a hemagglutinin (HA) epitope tag onto the C-terminus of RCMS editor constructs. HEK293T cells were cultured on six-well poly-d-lysine plates (Corning). When cells reached 80% confluency, each coverslip was transfected with 600 ng of HA-tagged editor plasmid and 400 ng of gRNA plasmid, using 4 μl of Hieff Trans™ Liposomal Transfection Reagent (YEASEN, China) following the manufacturer’s instructions. After 48 h, the culture medium was aspirated, and coverslips were washed once with PBS. Subsequently, cells were fixed by incubating them in 4% paraformaldehyde (Electron Microscopy Sciences) for 30 min, followed by washing with PBS three times. For staining, cells were treated with a rabbit anti-HA primary antibody (1:100, Sigma, H3663) in a blocking buffer (10% fetal bovine serum in PBS) for 1 h at 37°C. After three washes with PBS, cells were stained with a CoraLite488-conjugated goat anti-rabbit IgG (H + L) antibody (1:2000) (SA00013-2, Proteintech) in a blocking buffer for 30 min at 37°C. Olympus FV3000 confocal microscopy was used for imaging, with 405/488 nm of wavelength for excitation, using a 100× oil immersion lens.

### RNA decay assays

For assessing RNA stability, the life span of mRNA transcripts was determined following established procedures (53). Briefly, cells were treated with 10 μg/ml actinomycin D (Sigma–Aldrich, USA). After the cells, RNA was isolated using TRIzol for subsequent qRT-PCR analysis. The mRNA transcript life span was calculated, with 18S serving as the normalization reference for the data. The primers used in this study can be found in Supplementary Table S2.

### Wound healing assay

The wound healing assays were performed following a specified protocol (54). The HepG2 cells were seeded in six-well poly-d-lysine plates (Corning) with a wound healing insert, allowing cells to reach 100% confluency. Subsequently, a wound was created in the cell monolayer by employing a wound healing insert (Ibidi, Germany). After washing the cells with PBS buffer, they were further cultured for an additional 36 h. The cells were observed and imaged using a microscope.

### Statistical analysis

The data were subjected to statistical analysis using GraphPad Prism software 8.0.1 and presented as the mean ± standard deviation (SD). All statistical analyses are described in the figure legends. Statistical significance was determined at the probability value 0.05 (*P* < 0.05): *P < 0.05, **P < 0.01 and ***P < 0.001. The values represent biological replicates, and all the experiments are replicated at least three independent times.

## Results

### Design of programmable m^5^C editors

**Figure 1.**
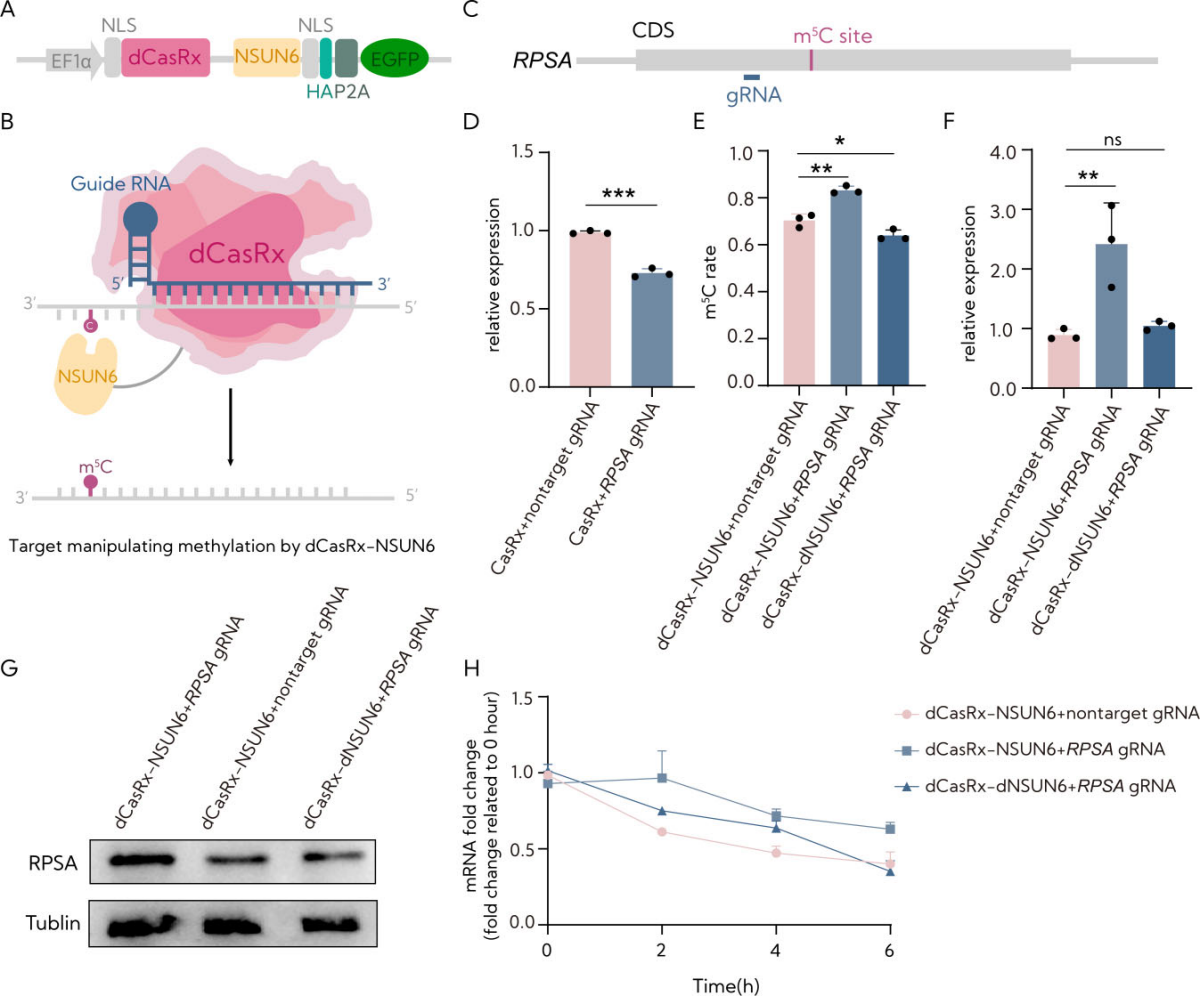
The validation of programmable site-specific m^5^C writer activity by *NSUN6* in HEK293T cells. (**A**) *NSUN6* methyltransferase was fused to dCasRx to create the RCMS dCasRx-NSUN6 editor. (**B**) The proposed strategy for RCMSs. This strategy involves employing a programmable RNA-binding protein like dCasRx fused with *NSUN6* methyltransferase to induce site-specific methylation changes from C to m^5^C by a manner of gRNA specified in a target transcript. (**C**) Schematic representation of the position of m^5^C site within *RPSA* mRNA and the targeted region by the gRNA. (**D**) Evaluation of the targeting efficiency of *RPSA* gRNA using CasRx by RT-qPCR (*n* = 3). (**E**) Analysis of the level of m^5^C ratio at the m^5^C site of *RPSA* after targeted m^5^C methylation by RCMS dCasRx-NSUN6 editor using HiTOM deep sequencing analysis (*n* = 3). (**F**) Assessment of mRNA levels of *RPSA* in HEK293T cells with dCasRx-NSUN6 or dCasRx-dNSUN6 combined with nontarget gRNA or *RPSA* gRNA (*n* = 3). (**G**) Analysis of protein levels of *RPSA* in HEK293T cells with dCasRx-NSUN6 or dCasRx-dNSUN6 combined with nontarget gRNA or RPSA gRNA. (**H**) Examination of *RPSA* levels in HEK293T cells treated with 10 mg/ml actinomycin D, a potent transcription inhibitor, at the indicated time points by RT-qPCR (*n* = 3). The ‘nontarget gRNA’ was used as a control for nontargeting. Error bars indicate the mean ± SD. *P < 0.05, **P < 0.01, ***P < 0.001 and n.s. indicates not significant by a two-tailed unpaired two-sample *t*-test.

**Figure 2.**
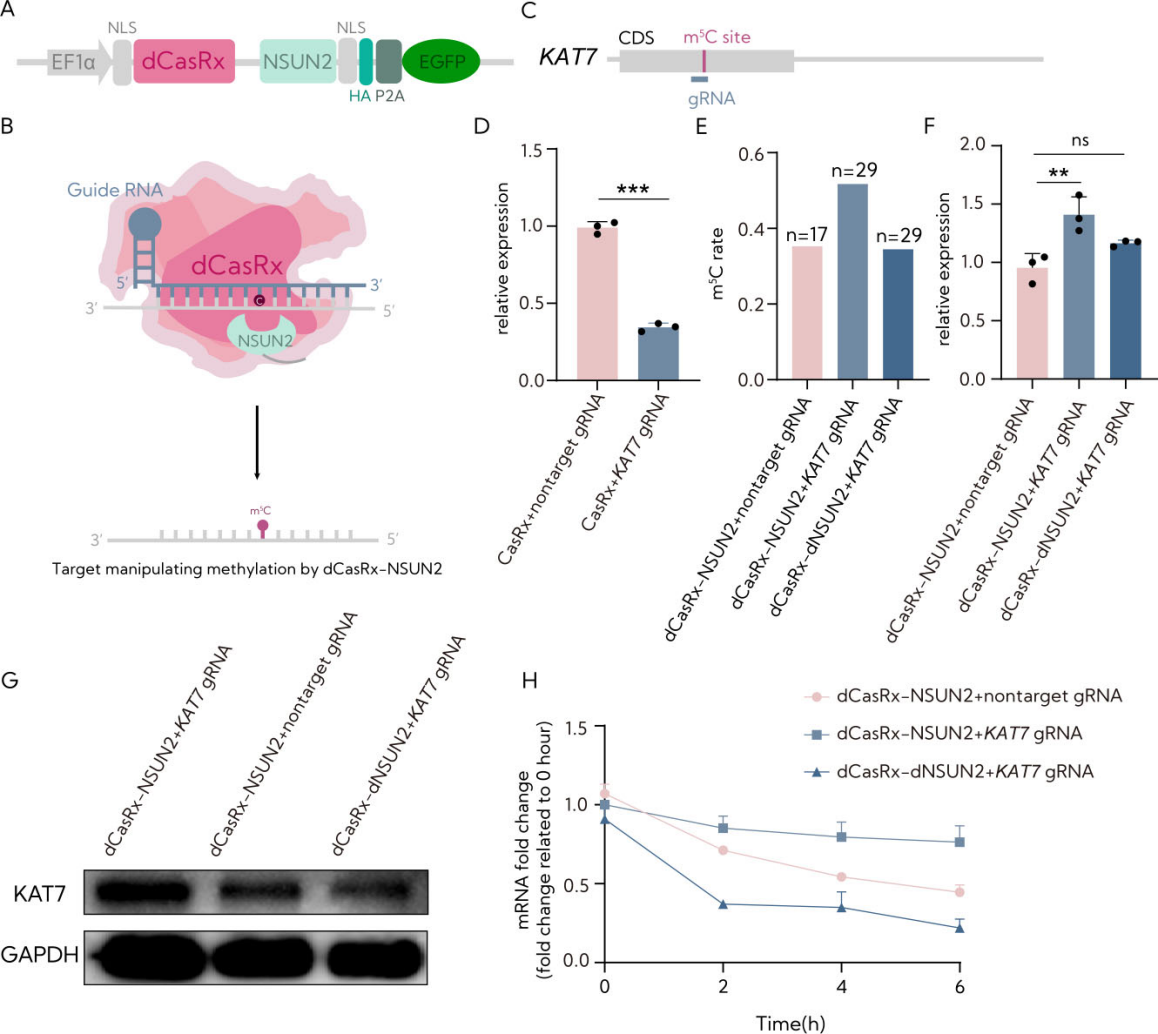
The methylation of endogenous transcripts by dCasRx-NSUN2 in human cells. (**A**) *NSUN2* methyltransferase was fused separately to dCasRx to generate the RCMS dCasRx-NSUN2 editor. (**B**) The proposed strategy for RCMSs using dCasRx with *NSUN2*. (**C**) Schematic representation of the position of m^5^C site within *KAT7* mRNA and the targeted region by the gRNA. (**D**) Measurement of the targeting efficiency of *KAT7* gRNA using CasRx by RT-qPCR (*n* = 3). (**E**) Quantification of the C-to-T mutation ratio at the m^5^C site of *KAT7* after targeted m^5^C methylation by RCMS dCasRx-NSUN2 editor, with the total tested numbers indicated above each bar [dCasRx-Tet2 CD + nontarget gRNA (6/17), dCasRx-Tet2 CD + *KAT7* gRNA (15/29), dCasRx-Tet2 CD + *KAT7* gRNA (10/29); *n*_1_/*n*_2_, *n*_1_ symbolizes the number of cytosines and *n*_2_ symbolizes the total tested numbers]. (**F**) Analysis of the mRNA levels of *KAT7* in HEK293T cells with dCasRx-NSUN2 combined with nontarget gRNA or *KAT7* gRNA (*n* = 3). (**G**) Assessment of the protein levels of *KAT7* in HEK293T cells with dCasRx-NSUN2 or dCasRx-dNSUN2 combined with nontarget gRNA or *KAT7* gRNA. (**H**) Examination of *KAT7* mRNA level in HEK293T cells treated with 10 mg/ml actinomycin D, a potent transcription inhibitor, at indicated time points by RT-qPCR (*n* = 3). The ‘nontarget gRNA’ was utilized for nontargeting. Error bars indicate the mean ± SD. *P < 0.05, **P < 0.01, ***P < 0.001 and n.s. stands for not significant by two-tailed unpaired two-sample *t*-test (*n* = 3).

**Figure 3.**
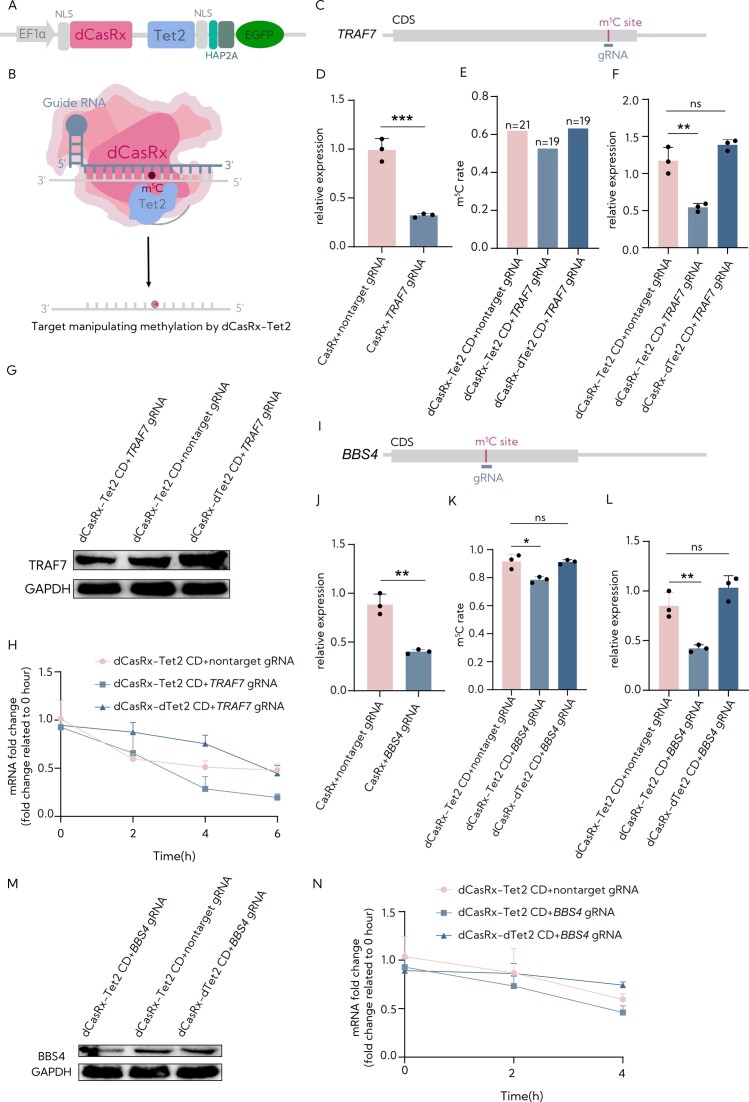
The targeted demethylation of mRNA transcripts by the RCMS eraser dCasRx-Tet2 CD. (**A**) Tet2 CD was fused separately to dCasRx to generate the RCMS dCasRx-Tet2 CD editor. (**B**) The proposed strategy for RCMSs using dCasRx with Tet2 CD. (**C**) Schematic representation of the m^5^C site within *TRAF7* (TNF receptor-associated factor 7) mRNA and the region targeted by the gRNA. (**D**) Measurement of the targeting efficiency of *TRAF7* gRNA using CasRx by RT-qPCR (*n* = 3). (**E**) Quantification of the C-to-T mutation ratio at the m^5^C site of *TRAF7* after targeted m^5^C methylation by RCMS dCasRx-Tet2 CD editor and the tested numbers are indicated above each bar [dCasRx-Tet2 CD + nontarget gRNA (13/21), dCasRx-Tet2 CD + *TRAF7* gRNA (10/19), dCasRx-Tet2 CD + *TRAF7* gRNA (12/19); *n*_1_/*n*_2_, *n*_1_ symbolizes the number of cytosines and *n*_2_ symbolizes the total tested numbers]. (**F**) The mRNA level of *TRAF7* in HEK293T cells with dCasRx-Tet2 CD combined with *TRAF7* gRNA or nontarget gRNA (*n* = 3). (**G**) The protein level of *TRAF7* in HEK293T cells with dCasRx-Tet2 CD combined with *TRAF7* gRNA or nontarget gRNA. (**H**) Examination of *TRAF7* mRNA levels in HEK293T cells treated with 10 mg/ml actinomycin D, a potent transcription inhibitor, at the indicated time points by RT-qPCR (*n* = 3). (**I**) Schematic representation of the position of m^5^C site within *BBS4* mRNA and the region targeted by the gRNA. (**J**) Measurement of the targeting efficiency of *BBS4* gRNA using CasRx by RT-qPCR (*n* = 3). (**K**) Quantification of the m^5^C ratio at the m^5^C site of *BBS4* after targeted m^5^C methylation by dCasRx-Tet2CD editor using deep sequence of HiTOM analysis (*n* = 3). (**L**) Analysis of the mRNA levels of *BBS4* in HEK293T cells with dCasRx-Tet2CD combined with *BBS4* gRNA or nontarget gRNA (*n* = 3). (**M**) Assessment of the protein level of *BBS4* in HEK293T cells with dCasRx-Tet2CD combined with *BBS4* gRNA or nontarget gRNA. (**N**) Examination of *BBS4* mRNA level in HEK293T cells treated with 10 mg/ml actinomycin D, a potent transcription inhibitor, at the indicated time points by RT-qPCR (*n* = 3). The ‘nontarget gRNA’ was used for nontargeting. Error bars indicate the mean ± SD. *P < 0.05, **P < 0.01, ***P < 0.001 and n.s. stands for not significant by two-tailed unpaired two-sample *t*-test.

To facilitate the study of RNA m^5^C modification, we fused m^5^C writers, *NSUN2*/*NSUN6*, and an eraser, Tet2 CD, with a catalytically inactive Cas13d variant from *Ruminococcus flavefaciens* XPD3002 (RfxCas13d, referred to as dCasRx hereafter) due to its reported RNA targeting activity in RNA base editors, to install or remove m^5^C at targeted sites with gRNAs. A previous study indicated a structural similarity to *Eubacterium siraeum* Cas13d (EsCas13d, PDB 6E9E) by cryo-electron microscopy, which shares 36% homology with CasRx, which revealed its C-terminal exposure (55) (Supplementary Figure S2A). Consequently, we linked dCasRx by its C-terminus to *NSUN6* (dCasRx-NSUN6) (Supplementary Figure S2B), NSUN2 (dCasRx-NSUN2) and Tet2 CD (dCasRx-Tet2 CD) using a flexible GSGGGGS linker. Given that human *NSUN6*, *NSUN2* and *Tet2* proteins are known to localize in various compartments (18,22,24,56), including the nucleus and cytosol, we attached two nuclear localization signals (NLS) to the RCMS editors, facilitating the import of the fusion protein into the cell nucleus (Figures 1A, 2A and 3A). These modified m^5^C editing complexes are designated as NLS-dCasRx-NSUN6-NLS-EGFP (dCasRx-NSUN6), NLS-dCasRx-NSUN2-NLS-EGFP (dCasRx-NSUN2) and NLS-dCasRx-Tet2 CD-NLS-EGFP (dCasRx-Tet2 CD). As depicted in Figures 1B, 2B and 3B, we proposed that the fusion of dCasRx with NSUN6/NSUN2/Tet2 could enable targeted m^5^C methylation and demethylation of specific transcripts.

### Validation of programmable site-specific m^5^C writer activity by NSUN6

To evaluate candidate RCMS constructs within a cellular environment, we performed western blotting to analyze the expression of dCasRx-NSUN6 and dCasRx-dead NSUN6 (dCasRx-dNSUN6). The results showed that dCasRx-NSUN6 and dCasRx-dNSUN6 were properly expressed in HEK293T cells (Supplementary Figure S3A). In addition, fluorescence microscopy data confirmed that the cells were GFP-positive (Supplementary Figure S3B and C), and flow cytometry displayed ∼50% of cells were GFP-positive (Supplementary Figure S3D). These data suggested that dCasRx-NSUN6 and dCasRx-dNSUN6 fusion proteins were successfully expressed in HEK293T cells. Moreover, an immunofluorescence assay further confirmed that NLS-tagged dCasRx-NSUN6 fusion protein mainly localized in the cytoplasm and nucleus of HEK293T cells (Supplementary Figure S3E).

It has been reported that NSUN6 methylated cytosine C234 within the coding region of *RPSA* mRNA displays substantial m^5^C methylation in m^5^C-seq databases (16,17). As proof of principle, we characterized the editing window of the RCMS dCasRx-NSUN6 editor by designing eight different gRNAs positioned at −150, −100, −50, −20, 0, +20, +50 and +100 nt surrounding the selected site (Supplementary Figure S4A). These gRNAs were co-transduced with dCasRx-NSUN6 and dCasRx-dNSUN6 into HEK293T cells. The results indicated varying responses to the dCasRx-NSUN6 editor to distinct gRNAs in the *RPSA* transcript and demonstrated high editing efficiency within the editing window from position −150 to −100 and 0 nt, with gRNA −150 nt exhibiting the highest efficiency compared to the nontargeted gRNA among eight gRNAs (Supplementary Figure S4B and C).

To investigate the ability of the dCasRx-NSUN6 editor in introducing m^5^C modifications on endogenous transcripts within HEK293T cells, based on the above results, we selected gRNA −150 nt to evaluate the efficiency of the dCasRx-NSUN6 editor at the m^5^C234 site of the *RPSA* transcript. As shown in Figure 1C, the co-transfection of the gRNA resulted in a significant reduction in *RPSA* levels when combined with CasRx (Figure 1D). Then, we targeted the *RPSA* transcript using the dCasRx-NSUN6 editor and the specific gRNA in HEK293T cells. The results demonstrated a significant increase in the m^5^C modification level at m^5^C234 within the *RPSA* mRNA induced by the dCasRx-NSUN6 editor compared to the nontargeting control and catalytically inactive control (Figure 1E), followed by an upregulation in mRNA and protein levels (Figure 1F and G). Furthermore, the heightened m^5^C rate caused a notable increase in the lifetime of *RPSA* transcripts (Figure 1H). Taken together, these results indicate that the fusion of dCasRx with a modified methyltransferase can selectively and efficiently introduce m^5^C modifications in exogenous RNAs within human cells.

### Methylation of endogenous transcripts by dCasRx-NSUN2 in human cells

*NSUN2* serves as a primary RNA m^5^C methyltransferase, acting on mRNA, tRNA (2,20,21,24) and some noncoding RNA (23) in eukaryotes. *NSUN2* is responsible for m^5^C methylation of type I mRNA (2,57) and at C38/C48/49/50 positions in tRNAs (20,21,24), which are situated adjacent to a downstream G-rich triplet motif. Thus, we constructed the plasmid encoding dCasRx-NSUN2 and dCasRx-dead NSUN2 (dCasRx-dNSUN2) by fusing human NSUN2 to dCasRx (Figure 2A and B) to further verify the site-specific installation m^5^C in a programmable manner. The successful expression and subcellular localization of the dCasRx-NSUN2 and dCasRx-dCasRx-dNSUN2 in the nucleus and cytoplasm are shown in Supplementary Figure S3A–E.

To determine the editing window of the dCasRx-NSUN2 editor, we targeted the transcript of lysine acetyltransferase 7 (*KAT7*), which was predicted by the m^5^C-Atlas (58). *KAT7* is associated with histone H4-specific acetyltransferase activity. Consistent with the above, we designed eight gRNAs, positioned at −150, −100, −50, −20, 0, +20, +50 and +100 nt relative to the m^5^C563 site of *KAT7*, tiling across an ∼300 bp region spanning the targeted site (Supplementary Figure S5A). The results showed that gRNA positioned from −50 to +20 nt upstream to the m^5^C563 site mediated robust m^5^C methylation and gRNA positioned at 0 nt displayed the highest effectiveness in elevating the m^5^C methylation level of the *KAT7* transcript upon co-expression of the certain gRNA with dCasRx-NSUN2 (Supplementary Figure S5B and C). These data indicated a significant correlation between the targeted m^5^C methylation efficiency of *KAT7* by dCasRx-NSUN2 and the distance of gRNA placement concerning the m^5^C site.

To further confirm the potential of dCasRx-NSUN2 in introducing m^5^C modifications on endogenous transcripts within HEK293T cells, we employed a 0 nt RNA specific to its coding region’s m^5^C563 site, which remarkably reduced the mRNA level (Figure 2C and D). We found that dCasRx-NSUN2 editor significantly increased the m^5^C level of KAT7 when targeted by the specific gRNA (by 1.5-fold, respectively) (Figure 2E) followed by an upregulation in the expression levels of KAT7 mRNA and protein (Figure 2F and G). Additionally, the lifetime of KAT7 transcripts was evidently increased by the dCasRx-NSUN2 editor (Figure 2H). Taken together, these results suggest that the RCMS dCasRx-NSUN2 editor effectively increases m^5^C methylation at the targeted RNA site.

### Validation of programmable site-specific m^5^C eraser activity by Tet2

Previous studies have demonstrated that Tet2 and ALKBH1 function as dioxygenase for both DNA and RNA, catalyzing the demethylation of m^5^C (26). To verify the potential of Tet2 to demethylate specific m^5^C sites, we fused the catalytic domain of mouse Tet2 with dCasRx (Figure 3A and B). The dCasRx-Tet2 CD and dCasRx-dTet2 CD were successfully expressed in cells and located within the nucleus and cytoplasm (Supplementary Figure S3A–E). Using RCMS eraser editor suite, we aimed to remove m^5^C modifications from endogenous transcripts in HEK293T cells. To achieve this, we designed eight gRNAs targeting the m^5^C808 site within the coding region of Bardet–Biedl syndrome 4 (*BBS4*), as shown in Supplementary Figure S6A. The results indicated that gRNAs positioned at −50, −20, 0, +20 and +50 nt effectively reduced the methylation level, with gRNA 0 nt exhibiting the highest efficiency of gRNA (Supplementary Figure S6B and C).

To further investigate the editing efficiency of dCasRx-Tet2 CD, our initial target was the m^5^C1683 site within the coding region of *TRAF7* mRNA, known for its high methylation in HEK293T cells (16,17). Subsequently, a gRNA spanning the m^5^C site was designed (Figure 3C), causing a reduction in the expression of mRNA when used with active CasRx (Figure 3D). To test whether Tet2 CD could lower levels of m^5^C at this specific site in human cells, we evaluated *TRAF7* methylation resulting from the dCasRx-Tet2CD construct using a specific gRNA designed for the *TRAF7* m^5^C1683 site.

Following transfection of these components, the m^5^C level at the *TRAF7* C1683 site notably decreased by ∼15% due to the action of RCMS dCasRx-Tet2CD editor with *TRAF7* gRNA (Figure 3E). Along with the removal of m^5^C, both mRNA and protein expression levels exhibited a significant decrease in the cells (Figure 3F and G). As shown in Figure 3H, the lifetime of *TRAF7* mRNA decreased when influenced by the dCasRx-Tet2CD editor with *TRAF7* gRNA. To further validate these results with other endogenous target transcripts, we targeted m^5^C808 within the coding region of *BBS4* (Figure 3I), known for its substantial methylation. Consistent with the above results, we detected a reduced *BBS4* methylation by dCasRx-Tet2CD in a manner dependent on an effective *BBS4*-targeting gRNA, accompanied by reduced levels of mRNA expression, protein expression and mRNA stability (Figure 3J–N). In addition, we designed a gRNA targeting m^5^C4968 within the coding region of calcineurin binding protein 1 (*CABIN1*) mRNA, which was effective in reducing *CABIN1* mRNA levels (Supplementary Figure S7A and B). As expected, the results revealed that the dCasRx-Tet2CD editor with the specific gRNA decreased the methylation of m^5^C4968 in *CABIN1* mRNA, resulting in a reduced life span of *CABIN1* mRNA (Supplementary Figure S7C–E). Therefore, these results showed that the dCasRx-Tet2CD editor can remove m^5^C in a programmable manner at specific sites.

### RCMSs induce programmable site-specific m^5^C levels in tRNA

The m^5^C modification has been identified across various RNA types, including not only mRNA but also tRNA and ribosomal RNA (59). In eukaryotes, tRNA and mRNA exhibit more prevalent m^5^C modifications compared to other RNA forms (60,61). However, most of the methylation modification editors [TRM (44), installing m^6^A; REMOVER (46), m^1^A editor; dCasRx-ALKBH5 (47), removing m^6^A; dm^6^ACRISPR (43), removing m^6^A] have scarcely been evaluated for their ability to regulate tRNA methylation levels. Therefore, we aimed to verify whether the RCMS editors could modulate the m^5^C levels in cells.

**Figure 4.**
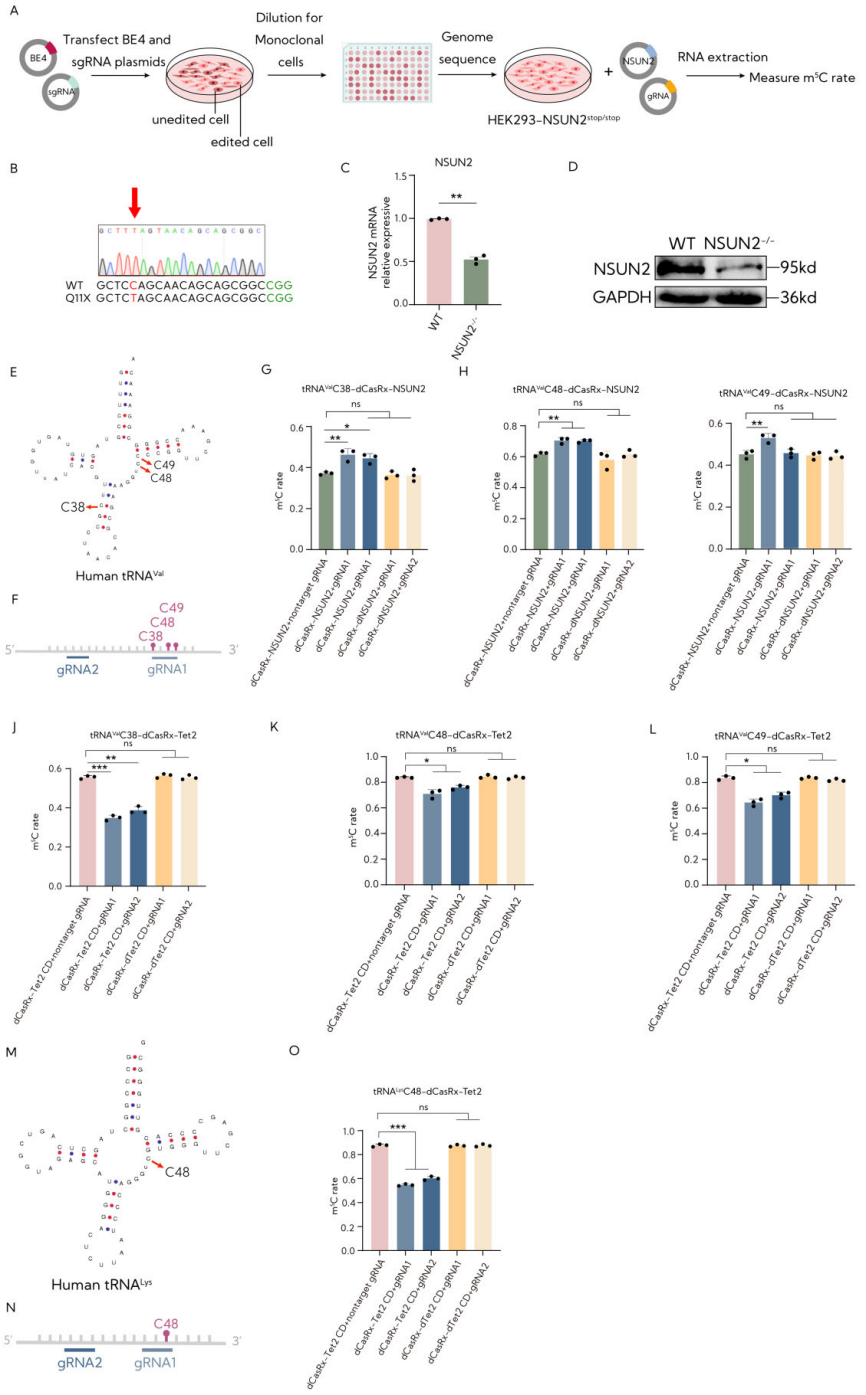
The targeted manipulation of tRNA transcript methylation and demethylation in HEK293T cells. (**A**) Schematic representation of the construction of NSUN2^−/−^ cell line. HEK293T cells were initially transfected with plasmids encoding a BE4max editor and gRNA targeting the NSUN2 gene. After transfection, cells were diluted and seeded into a 96-well plate for clonal selection and monoclonal cells were screened for the desired NSUN2^−/−^ genotype. (**B**) Sanger sequencing chromatogram of the NSUN2^−/−^ cell line in Gln11. (**C**) The mRNA levels of NSUN2 in NSUN2^−/−^ cell line (*n* = 3). (**D**) Western blot results of NSUN2 protein expression in WT cells and NSUN2^−/−^ cells. (**E**) Schematic of human tRNA^Val^ and three m^5^C sites marked with red arrows in tRNA^Val^. (**F**) Schematic representation of the position of the gRNAs in tRNA^Val^. (**G**) The m^5^C ratio in the m^5^C38 site of tRNA^Val^ after targeted m^5^C methylation by dCasRx-NSUN2 editor in NSUN2^−/−^ cell line using deep sequence HiTOM analysis (*n* = 3). (**H**) The m^5^C ratio in the m^5^C48 site of tRNA^Val^ after targeted m^5^C methylation by dCasRx-NSUN2 editor in NSUN2^−/−^ cell line using deep sequence HiTOM analysis (*n* = 3). (**I**) The m^5^C ratio in the m^5^C49 site of tRNA^Val^ after targeted m^5^C methylation by dCasRx-NSUN2 editor in NSUN2^−/−^ cell line using deep sequence of HiTOM analysis (*n* = 3). (**J**) The m^5^C ratio in the m^5^C38 site of tRNA^Val^ after targeted m^5^C methylation by dCasRx-Tet2 CD editor in HEK293T cells using deep sequence HiTOM analysis (*n* = 3). (**K**) The m^5^C ratio in the m^5^C48 site of tRNA^Val^ after targeted m^5^C methylation by dCasRx-Tet2 CD editor in HEK293T cells using deep sequence HiTOM analysis (*n* = 3). (**L**) The m^5^C ratio in the m^5^C49 site of tRNA^Val^ after targeted m^5^C methylation by dCasRx-Tet2 CD editor in HEK293T cells using deep sequence HiTOM analysis (*n* = 3). (**M**) Schematic of human tRNA^Lys^ highlighting the m^5^C48 site marked with a red arrow in tRNA^Lys^. (**N**) Schematic representation of the position of the gRNAs in tRNA^Lys^. (**O**) The m^5^C ratio in the m^5^C48 site of tRNA^Lys^ after targeted m^5^C methylation by dCasRx-Tet2 CD editor in HEK293T cells using deep sequence HiTOM analysis (*n* = 3). The ‘nontarget gRNA’ was used for nontargeting. Error bars indicate the mean ± SD. *P < 0.05, **P < 0.01, ***P < 0.001 and n.s. stands for not significant by two-tailed unpaired two-sample *t*-test.

Considering the ability of *NSUN2* to modify several positions (C34, C40, C48, C49 and C50) within different tRNAs (8,24,25), we initially constructed an *NSUN2* knockout HEK293T cell line, targeting Gln11 using the CBE4max system (Figure 4A). This genetic manipulation involved a single C-to-T conversion designed to induce a premature stop codon (p. Gln11stop) in exon 1 of *NSUN2* (Figure 4B), resulting in a considerable decrease in the expression of *NSUN2* (Figure 4C and D). Previous studies have indicated three m^5^C sites, m^5^C39, m^5^C48 and m^5^C49 (Figure 4E), in tRNA^Val^. To confirm the functionality of the dCasRx-NSUN2 construct with gRNAs in targeting tRNAs, we designed two gRNAs, one positioned at the m^5^C sites and the other located −7 nt upstream of the m^5^C38 site, both targeting tRNA^Val^ (Figure 4F). Subsequently, we co-transduced the targeted gRNAs for tRNA^Val^ and dCasRx-NSUN2 plasmids into the *NSUN2*^−/−^ cell line. Notably, the methylation levels of m^5^C39 and m^5^C48 significantly increased by ∼25% with both gRNAs and dCasRx-NSUN2 editor (Figure 4G and H). However, an increase in the m^5^C level was observed at the m^5^C49 site of tRNA^Val^ with gRNA1 rather than gRNA2, which is located in the targeted site (Figure 4I). We speculated that gRNAs positioned at the m^5^C sites effectively enhance the sensitivity of dCasRx-NSUN2 for tRNA methylation. Taken together, these results suggest that the RCMS dCasRx-NSUN2 editor leads to an elevation in m^5^C methylation at the targeted tRNA.

While previous studies have identified TET enzymes as RNA demethylases and demonstrated that overexpressing TETs could increase RNA hm^5^C levels in HEK293T cells (26), the specific targeting of tRNA by Tet2 has not been clarified. To further demonstrate the efficacy of eraser RCMS editors, our next objective was to determine whether Tet2 CD could target different RNA types. Thus, two different tRNAs, tRNA^Val^ and tRNA^Lys^, were selected for investigation (Figure 4E and M). The results suggested that gRNAs positioned at the m^5^C sites in tRNA^Val^ effectively sensitized dCasRx-Tet2 CD for tRNA demethylation, with gRNA1 exhibiting higher effectiveness compared to gRNA2 in tRNA^Val^ (Figure 4J–L). Similarly, the RCMS eraser dCasRx-Tet2 CD editor with gRNA1 exhibited effectiveness in tRNA^Lys^, although both gRNAs exhibited a decrease in methylation within the editor dCasRx-Tet2 CD in tRNA^Lys^ (Figure 4N and O). These results suggest that the eraser RCMS editor dCasRx-Tet2 CD displayed heightened activity when gRNAs were designed at the m^5^C sites on tRNA substrates, specifically decreasing the methylation level of m^5^C cytosines within the selected transcript. Overall, these findings establish that RCMS editors can induce the installation and removal of m^5^C levels in tRNAs.

### Off-target methylation by RCMS editors in human cells

**Figure 5.**
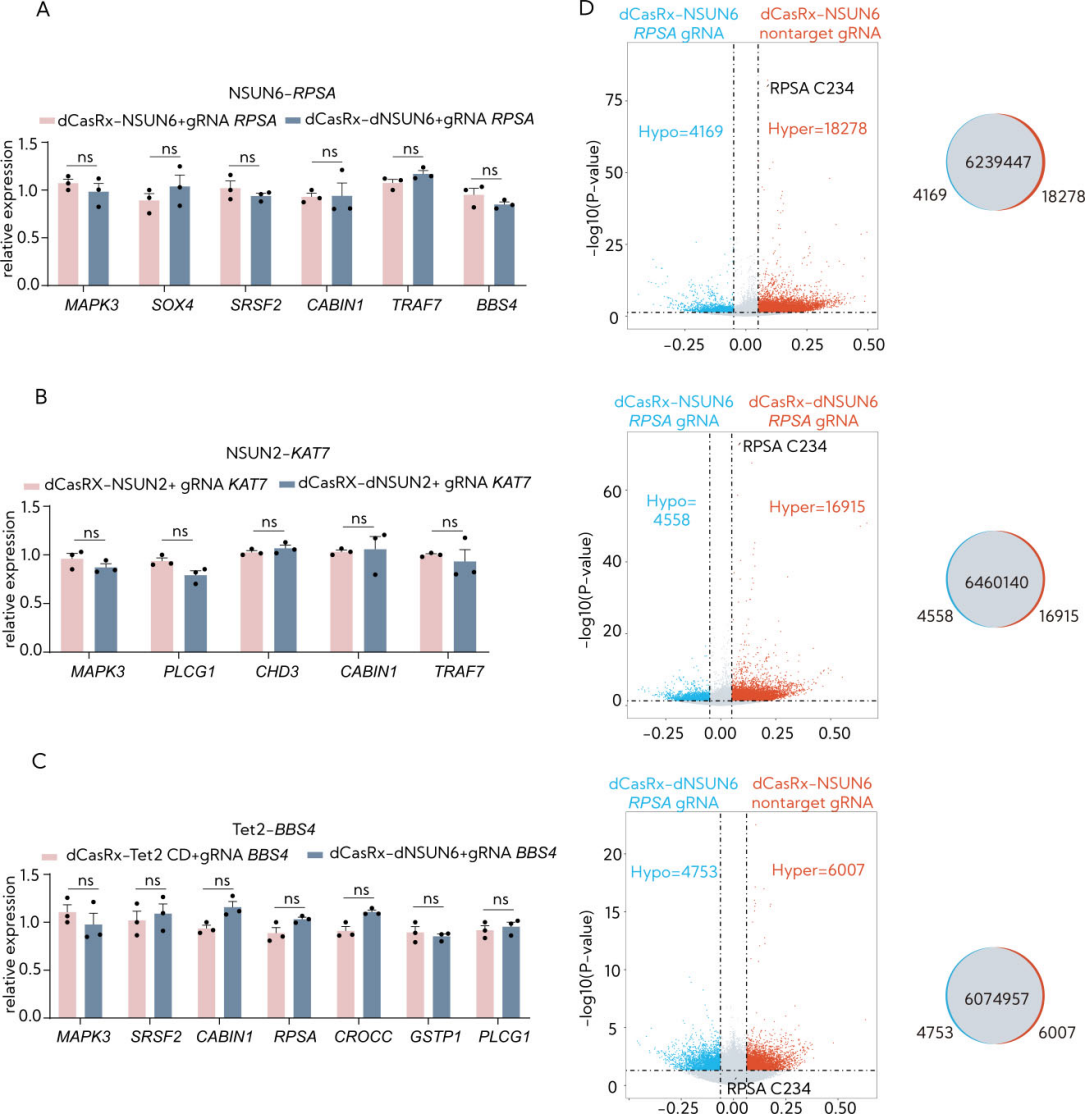
The specificity and off-target methylation of the RCMS dCasRx-NSUN6 editor. (**A**) Examination of the mRNA level of m^5^C-enriched transcripts after targeted m^5^C methylation of *RPSA* by RCMS dCasRx-NSUN6 editor (*n* = 3). (**B**) Examination of the mRNA level of m^5^C-enriched transcripts after targeted m^5^C demethylation of *KAT7* by RCMS dCasRx-NSUN2 editor (*n* = 3). (**C**) Examination of the mRNA level of m^5^C-enriched transcripts after targeted m^5^C demethylation of *BBS4* by RCMS dCasRx-Tet2 CD editor (*n* = 3). (**D**) Differential m^5^C enrichment of methylated sites in HEK293T cells transduced with dCasRx-NSUN6 or dCasRx-NSUN6 and *RPSA* targeted gRNA or nontarget gRNA. The top section represents differential methylation of m^5^C sites between the conditions indicated above, indicating differentially methylated sites with statistical significance (*P* < 0.05) are shown. The bottom section represents Venn diagrams depicting the overlap of all methylated m^5^C sites for the above-mentioned comparisons. BS-seq analysis was performed with three independent biological replicates. Nontarget gRNA was used for nontargeting. Error bars indicate the mean ± SD. *P < 0.05, **P < 0.01, ***P < 0.001 and n.s. stands for not significant by two-way analysis of variance followed by Dunnett’s multiple comparisons.

To assess the potential off-target effects of RCMS editors, we measured the changes in m^5^C-enriched mRNA at nontargeted m^5^C sites in HEK293T cells transduced with RCMS editors and specific gRNAs. Our findings indicated that cells transduced with both RCMS methylated editors and demethylated editors did not show any alterations in the mRNA expression levels at these nontargeted m^5^C sites (Figure 5A–C). This suggests a low risk of off-target editing associated with the dCasRx epitranscriptomic system.

To investigate whether the RCMS activity leads to substantial nonspecific methylation, we assessed the impact of the RCMS dCasRx-NSUN6 editor, guided by gRNA −150 nt targeting RPSA m^5^C234, on the overall m^5^C content using BSP-seq. In agreement with the above data, we observed increased m^5^C enrichment at C234 induced by dCasRx-NSUN6 with the *RPSA* gRNA −150 nt, while no such effect was seen with a nontargeting gRNA. Compared to an inactive control, dCasRx-NSUN6 with the specific gRNA increased the m^5^C level at 16 915 additional sites among the total of over 6 000 000 detected (0.3%) (Figure 5D). These findings indicate that the RCMS dCasRx-NSUN6 editor results in minimal off-target effects on the overall distribution of RNA methylation across the transcriptome.

### Programmable RNA m^5^C modification in HepG2 with RCMS

**Figure 6.**
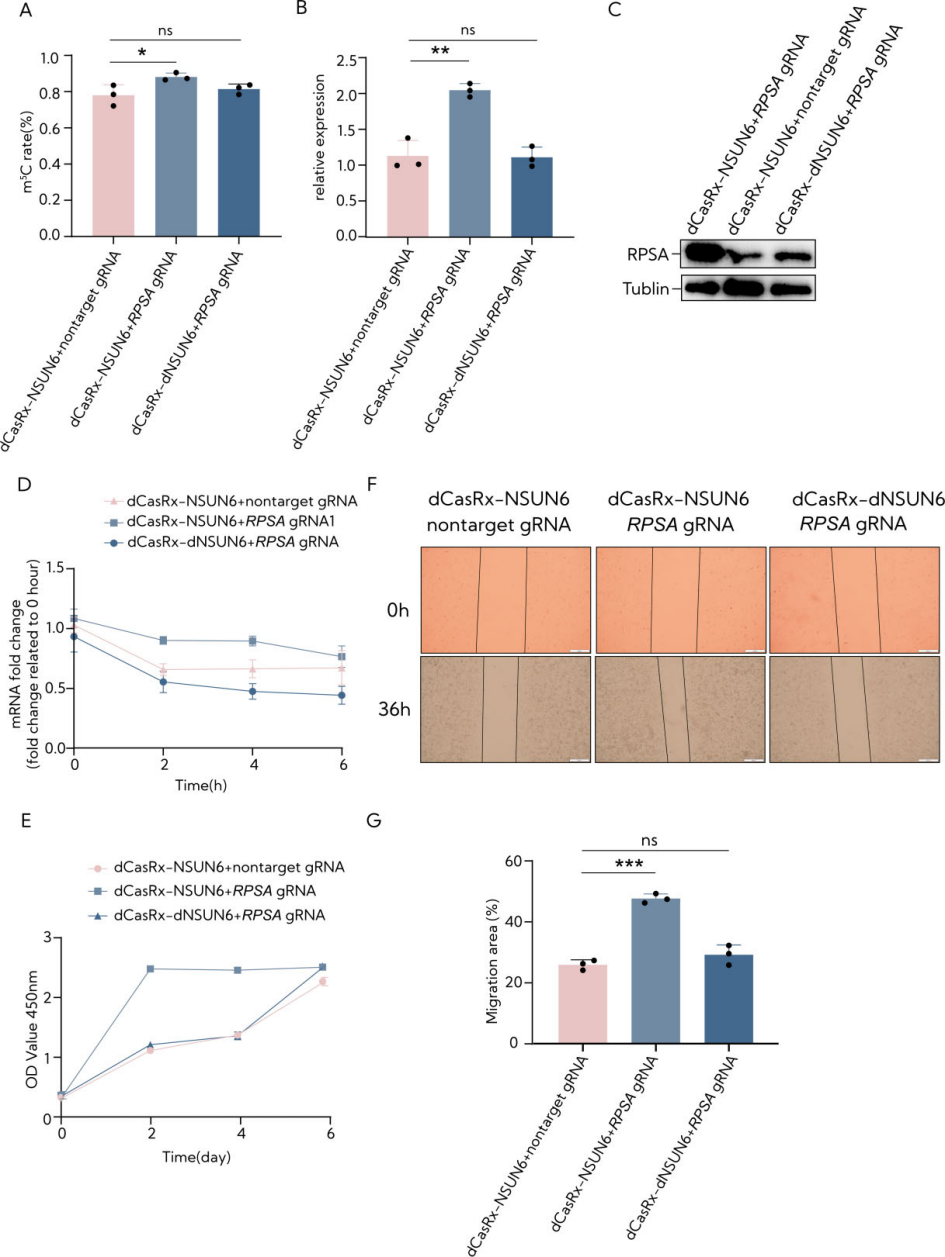
The *RPSA* m^5^C site modification in HepG2 cells using the RCMS dCasRx-NSUN6 editor. (**A**) The m^5^C ratio in the m^5^C234 site of *RPSA* targeted by RCMS dCasRx-NSUN6 editor along with *RPSA* gRNA −150 nt using deep sequence HiTOM analysis (*n* = 3). (**B**) The mRNA levels of *RPSA* in HEK293T cells transduced with dCasRx-NSUN6 or dCasRx-dNSUN6 combined with nontarget gRNA or *RPSA* gRNA −150 nt (*n* = 3). (**C**) The protein levels of *RPSA* in HEK293T cells transduced with dCasRx-NSUN6 or dCasRx-dNSUN6 combined with nontarget gRNA or *RPSA* gRNA −150 nt. (**D**) Examination of *RPSA* level in HEK293T cells treated with 10 mg/ml actinomycin D, a potent transcription inhibitor, at the indicated time points by RT-qPCR (*n* = 3). (**E**) Cell proliferation was determined by CCK8 assay in HepG2 cells transduced with dCasRx-NSUN6 or dCasRx-dNSUN6 and nontarget gRNA or *RPSA* gRNA −150 nt (*n* = 6). (**F**) Representative images of the wound healing assay of HepG2 cells with target methylation at *RPSA* using dCasRx-NSUN6 and *RPSA* gRNA −150 nt. The scratch measurements were recorded at 0 and 36 h after the initial scratch. Scale bars: 200 μm. (**G**) The statistical analysis of the wound healing assay (*n* = 3). Nontarget gRNA was used for nontargeting. Error bars indicate the mean ± SD. *P < 0.05, **P < 0.01, ***P < 0.001 and n.s. stands for not significant by two-tailed unpaired two-sample *t*-test.

The RCMS editors regulate methylation at specific sites, prompting us to investigate whether alterations in RNA methylation levels impact phenotypic differences. *RPSA*, ribosomal protein SA, encoding a high-affinity non-integrin family, laminin receptor 1, which is also called 67-kDa laminin receptor, 37-kDa laminin receptor precursor (37LRP) and p40 ribosome-associated protein, has been associated with the invasive and metastatic behavior of cancer cells upon its overexpression. Therefore, employing the gRNA −150 nt, we transduced RCMS dCasRx-NSUN6 via lentivirus delivery to target m^5^C234 within the RPSA CDS in HepG2 cells. We observed a significant increase in methylation level upon using the gRNA and dCasRx-NSUN6 editor (Figure 6A). Conversely, m^5^C modifications induced by dCasRx-NSUN6 editing significantly upregulated the relevant mRNA and protein levels (Figure 6B and C). To confirm that the effects of m^5^C editing were mediated by changes in degradation, we assessed mRNA stability, which is an improvement in stability associated with increased m^5^C levels (Figure 6D). Subsequently, we examined potential changes in cell proliferation using CCK8. The results indicated that the higher the methylation levels induced by the dCasRx-NSUN6 editor and targeted gRNA, the faster the cell proliferation (Figure 6E). In addition, wound healing assays illustrated significantly increased wound closure in cells subjected to dCasRx-NSUN6 with targeted gRNA compared to those treated with nontargeted gRNA or dCasRx-dNSUN6 with targeted gRNA (Figure 6F and G). These data signify that RCMS-mediated m^5^C methylation contributes to the stabilization of the target RNA and subsequently affects cell proliferation and migration processes.

**Figure 7.**
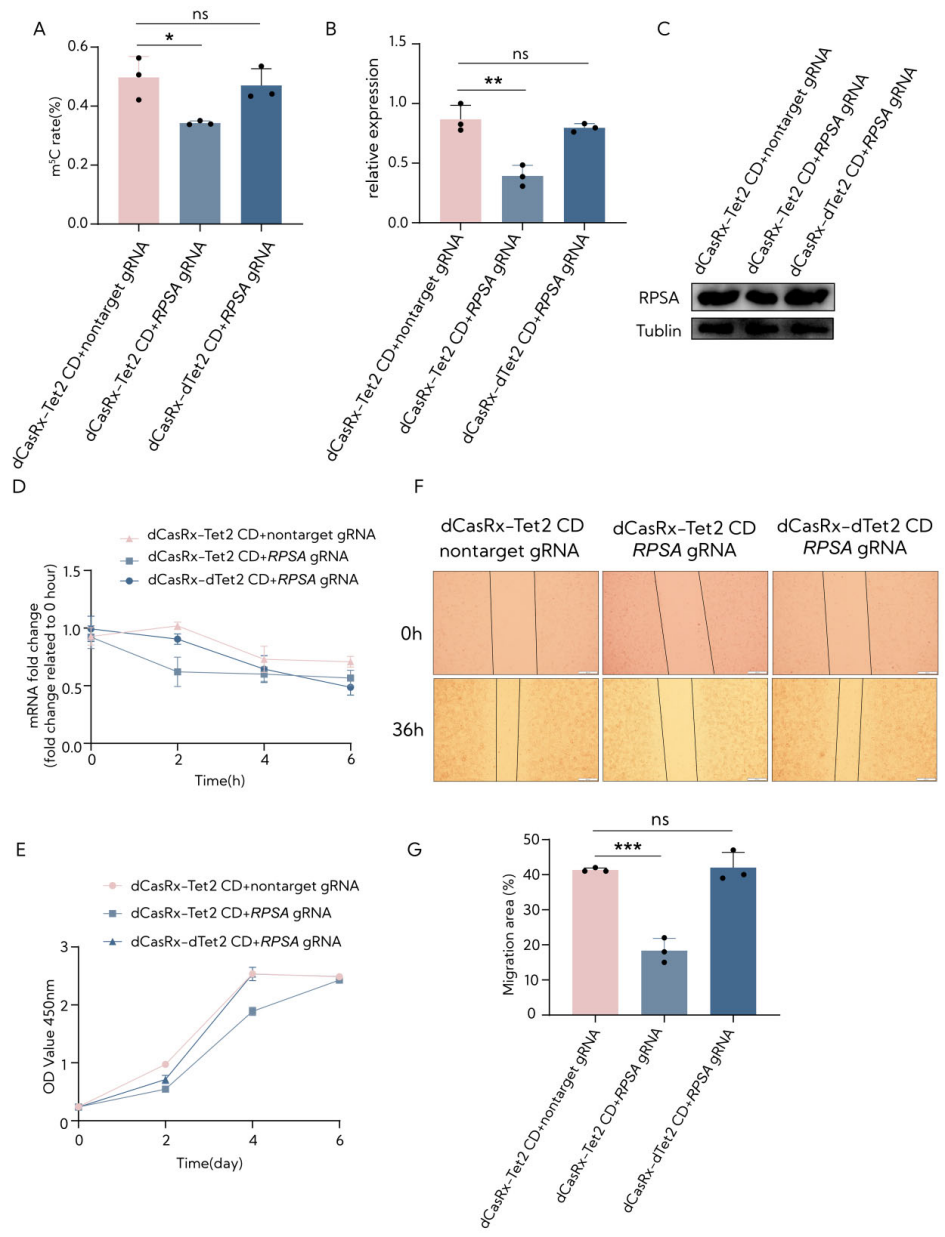
The *RPSA* m^5^C site modification in HepG2 cells using the RCMS dCasRx-NSUN6 editor. (**A**) The m^5^C ratio in the m^5^C234 site of RPSA targeted by RCMS dCasRx-Tet2 CD editor and *RPSA* gRNA 0 nt using deep sequence HiTOM analysis (*n* = 3). (**B**) The mRNA levels of RPSA in HEK293T cells transduced with dCasRx-Tet2 CD or dCas-dTet2 CD combined with nontarget gRNA or RPSA gRNA 0 nt (*n* = 3). (**C**) The protein levels of *RPSA* in HepG2 cells transduced with dCasRx-Tet2 CD or dCasRx-dTet2 CD combined with nontarget gRNA or *RPSA* gRNA 0 nt. (**D**) Examination of *RPSA* level in HepG2 cells treated with 10 mg/ml actinomycin D, a potent transcription inhibitor, at the indicated time points by RT-qPCR (*n* = 3). (**E**) Cell proliferation was determined by CCK8 assay in HepG2 cells transfected with dCasRx-Tet2 CD or dCasRx-dTet2 CD and nontarget gRNA or *RPSA* gRNA 0 nt (*n* = 6). (**F**) Representative images of the wound healing assay in HepG2 cells with target methylation at *RPSA* using dCasRx-Tet2 CD and *RPSA* gRNA 0 nt. The scratch measurements were recorded at 0 and 36 h after the initial scratch. Scale bars: 200 μm. (**G**) The statistical analysis of the wound healing assay. Nontarget gRNA was used for nontargeting. Error bars indicate the mean ± SD. *P < 0.05, **P < 0.01, ***P < 0.001 and n.s. stands for not significant by two-tailed unpaired two-sample *t*-test.

To further investigate whether the RCMS dCasRx-Tet2 CD editor could induce a decrease in m^5^C level and impact the invasive and metastatic phenotype in cancer cells, we transduced HepG2 cells with dCasRx-Tet2 CD with gRNA 0 nt or nontarget gRNA using a lentivirus system. The results showed that the methylation level was notably decreased in the effect of gRNA and dCasRx-Tet2 CD editor, along with a decrease in mRNA expression and protein levels (Figure 7A–C). Consistent with these findings, the removal of m^5^C led to accelerated mRNA degradation, resulting in decreased mRNA levels (Figure 7D). Assessments of cell proliferation and migration indicated that the reduction in m^5^C modification levels within *RPSA* mRNA remarkably inhibited the migration ability of HepG2 cells (Figure 7E–G). The results confirmed that the demethylation of *RPSA* mRNA using dCasRx-Tet2 CD guided by *RPSA* gRNA, as opposed to dCasRx-dTet2 CD, significantly suppressed the proliferation and migration of HepG2 cells. Overall, these results demonstrate the ability of gRNA target demethylation of *RPSA* mRNA by dCasRx-Tet2 CD to inhibit the progression of cell proliferation and migration.

## Discussion

In the study, we demonstrated the broad functionality of RCMSs in targeting various RNA targets through precise m^5^C installation or removal, influencing the stability of cellular transcripts in a methylation-dependent manner. Moreover, beyond their successful transfection into normal human cells, the dCasRx epitranscriptomic editors were effectively packaged into lentiviruses. This enabled the exploration of single m^5^C site functions in HepG2 cells, which were challenging to transfect through alternative methods. We demonstrated that m^5^C played a critical role in regulating *RPSA* mRNA expression, where changes in *RPSA* transcript levels impacted HepG2 cell proliferation. Overall, this study introduces the capability of utilizing CRISPR–Cas13 coupled with RNA methyltransferases and demethylases for targeted RNA methylation. This tool offers programmable manipulation of the epitranscriptome.

Within the Cas13 systems, most Cas13d proteins lack PFS restrictions, unlike other Cas13 proteins. They do not necessitate a protospacer adjacent motif and do not exhibit a specific motif preference at the editing sites. Notably, our study indicated that various RCMS editors appear to be influenced by the positioning of the gRNA concerning m^5^C sites. Specifically, in the case of dCasRx-NSUN6, gRNAs positioned −150 nt from the 5′ sequence of m^5^C sites appeared to enhance methylation efficiency. However, a reverse trend was observed in the other editors, namely dCasRx-NSUN2 and dCasRx-Tet2 CD, which exhibited higher effectiveness when gRNAs were located precisely on m^5^C sites. Consistent with these findings, previous studies have also observed that different fusions caused incongruent results using different gRNAs (43,44,46,62). Thus, we guess that this discrepancy resulted from different fusions, which possessed steric hindrances due to structural differences when fused with Cas enzymes.

Previous studies have identified numerous m^5^C sites within tRNA, notably situated on the variable loop, anticodon loop and T stem spanning positions (63). These sites are regulated by various methylases, including NSUN2 (25,44,64,65), NSUN3 (13,66), NSUN6 (18) and DNMT2 (21,67), and demethylases ALKBH1 and TET2. In our study using dCasRx epitranscriptomic editors, we demonstrated that these systems selectively targeted distinct tRNA substrates, modifying the m^5^C levels at specific sites. Consequently, these modifications affected the stability of tRNA, influencing translation efficiency. Our findings highlighted the efficacy of dCasRx-Tet2CD in reducing m^5^C levels across various tRNAs when guided by gRNAs located precisely on m^5^C sites. Collectively, these results underscore the utility of our RCMSs in unraveling previously ambiguous interactions to elucidate the causal relationships between m^5^C modifications and observed phenotypes.

In summary, this report marks the pioneering establishment of RCMS as a tool for targeted epitranscriptome engineering and explores its potential applications. Our findings demonstrate the broad functionality of RCMS, allowing for precise site-specific m^5^C installation or removal across various RNA targets. This alters the stability of cellular transcripts in a methylation-dependent manner. Our newly developed RCMSs have a unique potential to illuminate functional relationships between m^5^C and phenotype and advance basic research on m^5^C biology.

## Supplementary Material

gkae110_Supplemental_Files

## Data Availability

The data underlying this article are available in the Gene Expression Omnibus at https://www.ncbi.nlm.nih.gov/geo/, and can be accessed under GSE253659.
