# Supplementary material for: Programmable RNA 5-methylcytosine (m5C) modification of cellular RNAs by dCasRx conjugated methyltransferase and demethylase
Source: Nucleic Acids Res. 2024 Feb 15;52(6):2776–91. doi: 10.1093/nar/gkae110 (PMC11014266; doi:10.1093/nar/gkae110)
Supplement: gkae110_Supplemental_Files [file gkae110_supplemental_files.zip › Revised Supplement tables.pdf]

Supplementary Table 1 plamids protein sequences used in this study

Name	Protein sequence
dCasRx-NSUN6	MSPKKKRKVEASIEKKKSFAKGMGVKSTLVSGSKVYMTTFAEGSDARLEKIVEGDSIRSVNEGEAFSAEMA<br>DKNAGYKIGNAKFSHPKGYAVVANNPLYTGPVQQDMLGLKETLEKRYFGESADGNDNICIQVIHNILDIEKIL<br>AEYITNAAYAVNNISGLDKDIIGFGKFSTVYTYDEFKDPEHHRAAFNNNDKLINAIKAQYDEFDNFLDNPRLG<br>YFGQAFFSKEGRNYIINYGNECYDILALLSGLAHWVVANNEEESRISRTWLYNLDKNLDNEYISTLNYLYDRI<br>TNELTNSFSKNSAANVNYIAETLGINPAEFAEQYFRFSIMKEQKNLGFNITKLREVMLDRKDMSEIRKNHKVF<br>DSIRTKVYTMMDFVIYRYYIEEDAKVAAANKSLPDNEKSLSEKDIFVINLRGSFNDDQKDALYYDEANRIWR<br>KLENIMHNIKEFRGNKTREYKKKDAPRLPRILPAGRDVSAFSKLMYALTMFLDGKEINDLLTTLINKFDNIQS<br>FLKVMPLIGVNAKFVEEYAFFKDSAKIADELRLIKSFARMGEPIADARRAMYIDAIRILGTNLSYDELKALAD<br>TFSLDENGNKLKKGKHGMRNFIINNVISNKRFHYLIRYGDPAHLHEIAKNEAVVKFVLGRIADIQKKQGQNG<br>KNQIDRYYETCIGKDKGKSVSEKVDALTKIITGMNYDQFDKKRSVIEDTGRENAEREKFKKIISLYLTVIYHIL<br>KNIVNINARYVIGFHCVERDAQLYKEKGYDINLKKLEEKGFSSVTKLCAGIDETAPDKRKDVEKEMAERAKE<br>SIDSLESANPKLYANYIKYSDEKKAEEFTRQINREKAKTALNAYLRNTKWNVIIREDLLRIDNKTCTLFANKA<br>VALEVARYVHAYINDIAEVNSYFQLYHYIMQRIIMNERYEKSSGKVSEYFDAVNDEKKYNDRLLKLLCVPFG<br>YCIPRFKNLSIEALFDRNEAAKFDKEKKKVSGNSGSGGGGSMFHKRGKKNGKFSIVTALGKQEAERKFETLL<br>KHLSHPPSFTTVRVNTHLASVQHVKNLLLDELQKQFNGLSVPILQHPDLQDVLLIPVIGPRKNIKKQQCEAIV<br>GAQCGNAVLRGAHVYAPGIVSASQFMKAGDVISVYSDIKGKCKKGAKEFDGTKVFLGNGISELSRKEIFSGL<br>PELKGMGIRMTEPVYLSPSFDSVLPRYLFLQNLPSALVSHVLNPQPGEKILDLCAAPGGKTTHIAALMHDQGE<br>VIALDKIFNKVEKIKQNALLLGLNSIRAFCFDGTKAVKLDMVEDTEGEPPFLPESFDRILLDAPCSGMGQRPN<br>MACTWSVKEVASYQPLQRKLFTAAVQLLKPEGVLVYSTCTITLAENEEQVAWALTKFPCLQLQPQEPQIGG<br>EGMRGAGLSCEQLKQLQRFDPSAVPLPDTDMDSLREARREDMLRLANKDSIGFFIAKFVKCKSTGSGPKKKR<br>KV

dCasRx-NSUN2	MSPKKKRKVEASIEKKKSFAKGMGVKSTLVSGSKVYMTTFAEGSDARLEKIVEGDSIRSVNEGEAFSAEMA<br>DKNAGYKIGNAKFSHPKGYAVVANNPLYTGPVQQDMLGLKETLEKRYFGESADGNDNICIQVIHNILDIEKIL<br>AEYITNAAYAVNNISGLDKDIIGFGKFSTVYTYDEFKDPEHHRAAFNNNDKLINAIKAQYDEFDNFLDNPRLG<br>YFGQAFFSKEGRNYIINYGNECYDILALLSGLAHWVVANNEEESRISRTWLYNLDKNLDNEYISTLNYLYDRI<br>TNELTNSFSKNSAANVNYIAETLGINPAEFAEQYFRFSIMKEQKNLGFNITKLREVMLDRKDMSEIRKNHKVF<br>DSIRTKVYTMMDFVIYRYYIEEDAKVAAANKSLPDNEKSLSEKDIFVINLRGSFNDDQKDALYYDEANRIWR<br>KLENIMHNIKEFRGNKTREYKKKDAPRLPRILPAGRDVSAFSKLMYALTMFLDGKEINDLLTTLINKFDNIQS<br>FLKVMPLIGVNAKFVEEYAFFKDSAKIADELRLIKSFARMGEPIADARRAMYIDAIRILGTNLSYDELKALAD<br>TFSLDENGNKLKKGKHGMRNFIINNVISNKRFHYLIRYGDPAHLHEIAKNEAVVKFVLGRIADIQKKQGQNG<br>KNQIDRYYETCIGKDKGKSVSEKVDALTKIITGMNYDQFDKKRSVIEDTGRENAEREKFKKIISLYLTVIYHIL<br>KNIVNINARYVIGFHCVERDAQLYKEKGYDINLKKLEEKGFSSVTKLCAGIDETAPDKRKDVEKEMAERAKE<br>SIDSLESANPKLYANYIKYSDEKKAEEFTRQINREKAKTALNAYLRNTKWNVIIREDLLRIDNKTCTLFANKA<br>VALEVARYVHAYINDIAEVNSYFQLYHYIMQRIIMNERYEKSSGKVSEYFDAVNDEKKYNDRLLKLLCVPFG<br>YCIPRFKNLSIEALFDRNEAAKFDKEKKKVSGNSGSGGGGSMGRRSRGRRLQQQQRPEDAEDGAEGGGKRG<br>EAGWEGGYPEIVKENKLFEHYYQELKIVPEGEWGQFMDALREPLPATLRITGYKSHAKEILHCLKNKYFKEL<br>EDLEVDGQKVEVPQPLSWYPEELAWHTNLSRKILRKSPHLEKFHQFLVSETESGNISRQEAVSMIPPLLLNVR<br>PHHKILDMCAAPGSKTTQLIEMLHADMNVPFPEGFVIANDVDNKRCYLLVHQAKRLSSPCIMVVNHDASSIP<br>RLQIDVDGRKEILFYDRILCDVPCSGDGTMRKNIDVWKKWTTLNSLQLHGLQLRIATRGAEQLAEGGRMVY<br>STCSLNPIEDEAVIASLLEKSEGALELADVSNELPGLKWMPGITQWKVMTKDGQWFTDWDAVPHSRHTQIR<br>PTMFPPKDPEKLQAMHLERCLRILPHHQNTGGFFVAVLVKKSSMPWNKRQPKLQGKSAETRESTQLSPADL<br>TEGKPTDPSKLESPSFTGTGDTEIAHATEDLENNGSKKDGVCGPPPSKKMKLFGFKEDPFVFIPEDDPLFPPIE<br>KFYALDPSFPRMNLLTRTTEGKKRQLYMVSKELRNVLLNNSEKMKVINTGIKVWCRNNSGEEFDCAFRLAQ<br>EGIYTLYPFINSRIITVSMEDVKILLTQENPFFRKLSSETYSQAKDLAKGSIVLKYEPDSANPDALQCPIVLCGW<br>RGKASIRTFVPKNERLHYLRMMGLEVLGEKKKEGVILTNESAASTGQPDNDVTEGQRAGEPNSPDAEEANSP<br>DVTAGCDPAGVHPPRGSGPKKKRKV
dCasRx-Tet2 CD	MSPKKKRKVEASIEKKKSFAKGMGVKSTLVSGSKVYMTTFAEGSDARLEKIVEGDSIRSVNEGEAFSAEMA<br>DKNAGYKIGNAKFSHPKGYAVVANNPLYTGPVQQDMLGLKETLEKRYFGESADGNDNICIQVIHNILDIEKIL<br>AEYITNAAYAVNNISGLDKDIIGFGKFSTVYTYDEFKDPEHHRAAFNNNDKLINAIKAQYDEFDNFLDNPRLG<br>YFGQAFFSKEGRNYIINYGNECYDILALLSGLAHWVVANNEEESRISRTWLYNLDKNLDNEYISTLNYLYDRI<br>TNELTNSFSKNSAANVNYIAETLGINPAEFAEQYFRFSIMKEQKNLGFNITKLREVMLDRKDMSEIRKNHKVF<br>DSIRTKVYTMMDFVIYRYYIEEDAKVAAANKSLPDNEKSLSEKDIFVINLRGSFNDDQKDALYYDEANRIWR<br>KLENIMHNIKEFRGNKTREYKKKDAPRLPRILPAGRDVSAFSKLMYALTMFLDGKEINDLLTTLINKFDNIQS<br>FLKVMPLIGVNAKFVEEYAFFKDSAKIADELRLIKSFARMGEPIADARRAMYIDAIRILGTNLSYDELKALAD<br>TFSLDENGNKLKKGKHGMRNFIINNVISNKRFHYLIRYGDPAHLHEIAKNEAVVKFVLGRIADIQKKQGQNG<br>KNQIDRYYETCIGKDKGKSVSEKVDALTKIITGMNYDQFDKKRSVIEDTGRENAEREKFKKIISLYLTVIYHIL<br>KNIVNINARYVIGFHCVERDAQLYKEKGYDINLKKLEEKGFSSVTKLCAGIDETAPDKRKDVEKEMAERAKE<br>SIDSLESANPKLYANYIKYSDEKKAEEFTRQINREKAKTALNAYLRNTKWNVIIREDLLRIDNKTCTLFANKA<br>VALEVARYVHAYINDIAEVNSYFQLYHYIMQRIIMNERYEKSSGKVSEYFDAVNDEKKYNDRLLKLLCVPFG<br>YCIPRFKNLSIEALFDRNEAAKFDKEKKKVSGNSGSGGGGSQSQNGKCEGCNPDKDEAPYYTHLGAGPDVA<br>AIRTLMEERYGEKGKAIRIEKVIYTGKEGKSSQGCPIAKWVYRRSSEEEKLLCLVRVRPNHTCETAVMVIAIM<br>LWDGIPKLLASELYSELTDILGKCGICTNRRCSQNETKKKQSPPRNCCCQGENPETCGASFSFGCSWSMYYN<br>GCKFARSKKPRKFRLHGAEPKEEERLGSHLQNLATVIAPIYKKLAPDAYNNQVEFEHQAPDCCLGLKEGRPF<br>SGVTACLDFSAHSHRDQQNMPNGSTVVVTLNREDNREVGAKPEDEQFHVLPMYIIAPEDEFGSTEGQEKKIR<br>MGSIEVLQSFRRRRVIRIGELPKSCKKKAEPKKAKTKKAARKRSSLENCSSRTEKGKSSSHTKLMENASHMK<br>QMTAQPQLSGPVIRQPPTLQRHLQQGQRPQQPQPPQPQPQTTPQPQPQPQHIMPGNSQSVGSHCSGSTSVYTR<br>QPTPHSPYPSSAHTSDIYGDTNHVNFYPTSSHASGSYLNPSNYMNPYLGLLNQNNQYAPFPYNGSVPVDNGS<br>PFLGSYSPQAQSRDLHRYPNQDHLTNQNLPPIHTLHQQTFGDSPSKYLSYGNQNMQRDAFTTNSTLKPNVHH<br>LATFSPYPTPKMDSHFMGAASRSPYSHPHTDYKTSEHHLPSHTIYSYTAAASGSSSSHAFHNKENDNIANGLS<br>RVLPGFNHDRTASAQELLYSLTGSSQEKQPEVSGQDAAAVQEIEYWSDSEHNFQDPCIGGVAIAPTHGSILIEC<br>AKCEVHATTKVNDPDRNHPTRISLVLYRHKNLFLPKHCLALWEAKMAEKARKEEECGKNGSDHVSQKNHG<br>KQEKREPTGPQEPSYLRFIQSLAENTGSVTTDSTVTTSPYAFTQVTGPYNTFVGSGPKKKRKV

Supplementary Table 2 Primers used in the study

Primers for cloning RCMS editors expression plasmids

**Name**	**Sequence**
**dCasRx-NSUN6**	Frament-F:ggatccggaggaggtggaagcatgttccataagcgtg
	Frament-R:tttttcttaggtccggatcctgtgcttttgcattttacaa
	Vector-F:ggatccggacctaagaaaaagagga
	Vector-R:gcttccacctcctccggatccggaattgccggaca
**dCasRx-Tet2 CD**	Frament-F:ggaggaggtggaagccaaagtcagaatggcaaatgtgaaggat
	Frament-R:cttaggtccggatcctacaaatgtgttgtaaggccctgtgac
	Vector-F:ggatccggacctaagaaaaagaggaaggtggcggccgct
	Vector-R:gcttccacctcctccggatccggaattgccggacacc

Primers for cloning lentivirus plasmids

**Name**	**Sequence**
**pCDH-CMV-dCasRx-NSUN6-puro**	Frament-F:tagagctagcgaattatgagccccaagaagaagagaaag
	Frament-R:agatccttcgcggccTTActtgtacagctcgtccatgccg
**pCDH-CMV-dCasRx-NSUN2-puro**	Frament-F:catagaagattctagatgagccccaagaagaagagaaag
	Frament-R:atttaaattcgaattTTAagcgtaatctggaacatcgtatgg
**pCDH-CMV-dCasRx-Tet2 CD-puro**	Frament-F:catagaagattctagatgagccccaagaagaagagaaag
	Frament-R:gcttccacctcctccggatccggaattgccggacacc

QPCR primers used in the study

RPSA-E4-5-qPCR-F	cctaccattgcgctgtgtaa
RPSA-E4-5-qPCR-R	caccacatcaaacccactga
KAT7-E2-3-qPCR-F	cctcgaactccaactggaaatg
KAT7-E2-3-qPCR-R	atgtccttggcaatgctctc
CABIN1-E28-29-qPCR-F	ggtgtcctccatgcttcag
CABIN1-E28-29-qPCR-R	ctcgctcagcgtgtcttc
TRAF7-E17-18-qPCR-F	tctggtggcagcgtcta
TRAF7-E17-18-qPCR-R	ttggactcaatgtcccacac
BBS4-qPCR-F	gttgtggcttgtgctgttc
BBS4-qPCR-R	ctcgtttcaggcagctgat
NSUN2-qPCR-F	ccgagggtggtggaaag
NSUN2-qPCR-R	tcttgagctcctggtagtagt
GAPDH-qPCR-F	gtcaacggatttggtcgta
GAPDH-qPCR-R	gtagttgaggtcaatgaag
18S-qPCR-F	atcctcagtgagttctcccg
18S-qPCR-R	ctttgccatcactgccatta

Primers for BSP PCR

RPSA-F	taaattttaagaggatttgggagaag
RPSA-R	caaccctaaaatcaataaccacaaaaaaccata
EEF2-F	ttaaaagttgtgattttaaaggtttttt
EEF2-R	acccacaatattcccacaaaac
KAT7-F	aggatttaggtagtgatttttt
KAT7-R	aacatcctttcttctatctac
CABIN1-F	tgttgtttaaggtgttggtttagtt
CABIN1-R	accttcacaataaaaataaaaaccc
TRAF7-F	gttttggtggttgtttagagttattt
TRAF7-R	actccttaaactcaatatcccacac
BBS4-F	gtttttattaaatatagagt
BBS4-R	cttcttrccaaaaaaacaca
tRNA Val AAC-F	gtttttgtagtgtagtggttat

tRNA Val AAC-R	tatttccacccaatttcaaacc
tRNA-Lys-CTT-F	gtttggttagtttagtt
tRNA-Lys-CTT-R	cacgacaccagttga

Primers for RNA BSP deep Bridging sequence

Bridging Forward primer	ggagtgagtacggtgtgc
Bridging Reverse primer	gagttggatgctggatgg

Supplementary Table 3 gRNA spacer sequences for M5C

Name	Spacer sequence
RPSA-gRNA -150-F	aaacgtgccacctaagtgggttcctgctgcaagg
RPSA-gRNA -150-R	aaaaccttgcagcaggaacccacttaggtggcac
RPSA-gRNA -100-F	aaacgcatcacttttccttttatagatgtactgtt
RPSA-gRNA -100-R	aaaaaacagtacatctataaaaggaaaagtgatgc
RPSA-gRNA -50-F	aaacgtgccagcagaagcttctcccaggtcctctt
RPSA-gRNA -50-R	aaaaaagaggacctgggagaagcttctgctggcac
RPSA-gRNA -20-F	aaacgagggttttcaatggcaacaattgcacgagc
RPSA-gRNA -20-R	aaaagctcgtgcaattgttgccattgaaaaccctc
RPSA-gRNA 0-F	aaacgccagtattcctggaggatataacactgac
RPSA-gRNA 0-R	aaaagtcagtgttatatcctccaggaatactggc
RPSA-gRNA +20-F	aaacgccagtggcagcagcaaacttcagcacagcc
RPSA-gRNA +20-R	aaaaggctgtgctgaagtttgctgctgccactggc
RPSA-gRNA +50-F	aaacggagtgaagcggccagcaattggagtggct
RPSA-gRNA +50-R	aaaaagccactccaattgctggccgcttcactcc
RPSA-gRNA +100-F	aaacgaagccgtggctcccggaaggctgcctgga
RPSA-gRNA +100-R	aaaatccaggcagccttccgggagccacggcttc
KAT7-gRNA -150-F	aaacgactcagaagaaggcgcatttccagttggag
KAT7-gRNA -150-R	aaaactccaactggaaatgcgccttcttctgagtc
KAT7-gRNA -100-F	aaacggcaatgctctcatcgtgagatacattggg
KAT7-gRNA -100-R	aaaacccaatgtatctcacgatgagagcattgcc
KAT7-gRNA -50-F	aaacgttggggcgatgagagagatcactgcctgag
KAT7-gRNA -50-R	aaaactcaggcagtgatctctctcatcgccccaac
KAT7-gRNA -20-F	aaacgaagttgtagctttcatggaagcgacggcgc
KAT7-gRNA -20-R	aaaagcgccgtcgcttccatgaaagctacaacttc
KAT7-gRNA 0-F	aaacgagagttacagcctggtgtaggacacttca
KAT7-gRNA 0-R	aaaatgaagtgtcctacaccaggctgtaactctc
KAT7-gRNA +20-F	aaacgagaaatgtctctcatgttttcctgtaaggt
KAT7-gRNA +20-R	aaaaaccttacaggaaaacatgagagacatttctc
KAT7-gRNA +50-F	aaacggttatgatacagtgggcatcctgagatgg
KAT7-gRNA +50-R	aaaaccatctcaggatgcccactgtatcataacc
KAT7-gRNA +100-F	aaactatctgcttatcccggctctgtgctctcac
KAT7-gRNA +100-R	aaaagtgagagcacagagccgggataagcagatac
BBS4-gRNA -150-F	aaacgtctggtaaatgccgagctgtaagtagagta
BBS4-gRNA -150-R	aaaatactctacttacagctcggcatttaccagac
BBS4-gRNA -100-F	aaacgcttgtagttggtagggtcataagtcagtgc
BBS4-gRNA -100-R	aaaacaaagcctcctgcctatacgggcagctcccc
BBS4-gRNA -50-F	aaacgtcaaagtccccgtgggtctgcatcatgctg
BBS4-gRNA -50-R	aaaacagcatgatgcagacccacggggactttgac
BBS4-gRNA -20-F	aaacggccacaactctgtatttggtgagggcaaca

BBS4-gRNA -20-R	aaaatgttgccctcaccaaatacagagttgtggcc
BBS4-gRNA 0-F	aaacgttattccagagtggaggactttctggaaca
BBS4-gRNA 0-R	aaaatgttccagaaagtcctccactctggaataac
BBS4-gRNA +20-F	aaacgcatatttcttcttgccaaagaaacacattc
BBS4-gRNA +20-R	aaaagaatgtgtttctttggcaagaagaaatatgc
BBS4-gRNA +50-F	aaacgtggctcgtttcaggcagctgatggccgcca
BBS4-gRNA +50-R	aaaatggctcgtttcaggcagctgatggccgccac
BBS4-gRNA +100-F	aaacgacaaggcccaaattatacagaatcttcca
BBS4-gRNA +100-R	aaaatggaagattctgtataatttgggccttgtc
TRAF7-gRNA-F	aaacgtgtcacagcaatggagtagacgctgccacc
TRAF7-gRNA-R	aaaaggtggcagcgtctactccattgctgtgacac
CABIN1-gRNA-F	aaacgccgctgaagcatggaggacaccttcagcag
CABIN1-gRNA-R	aaaactgctgaaggtgtcctccatgcttcagcggc
tRNA-Val-gRNA1-F	aaacgtttcgaaccggggacctttcgcgtgttagg
tRNA-Val-gRNA1-R	aaaacctaacacgcgaaaggtccccggttcgaaa
tRNA-Val-gRNA2-F	aaacgcgaacgtgataaccactacactacggaaac
tRNA-Val-gRNA2-R	aaaagtttccgtagtgtagtggttatcacgttcgc
tRNA-Lys-gRNA1-F	aaacgtggggctcgaacccacgaccctgggattaa
tRNA-Lys-gRNA1-R	aaaattaatcccagggtcgtgggttcgagccccac
tRNA-Lys-gRNA2-F	aaacgtcccatgctctaccgactgagctagccgg
tRNA-Lys-gRNA2-R	aaaaccggctagctcagtcggtagagcatgggac
