# Supplementary material for: Programmable RNA 5-methylcytosine (m5C) modification of cellular RNAs by dCasRx conjugated methyltransferase and demethylase
Source: Nucleic Acids Res. 2024 Feb 15;52(6):2776–91. doi: 10.1093/nar/gkae110 (PMC11014266; doi:10.1093/nar/gkae110)
Supplement: gkae110_Supplemental_Files [file gkae110_supplemental_files.zip › Supplement.pdf]

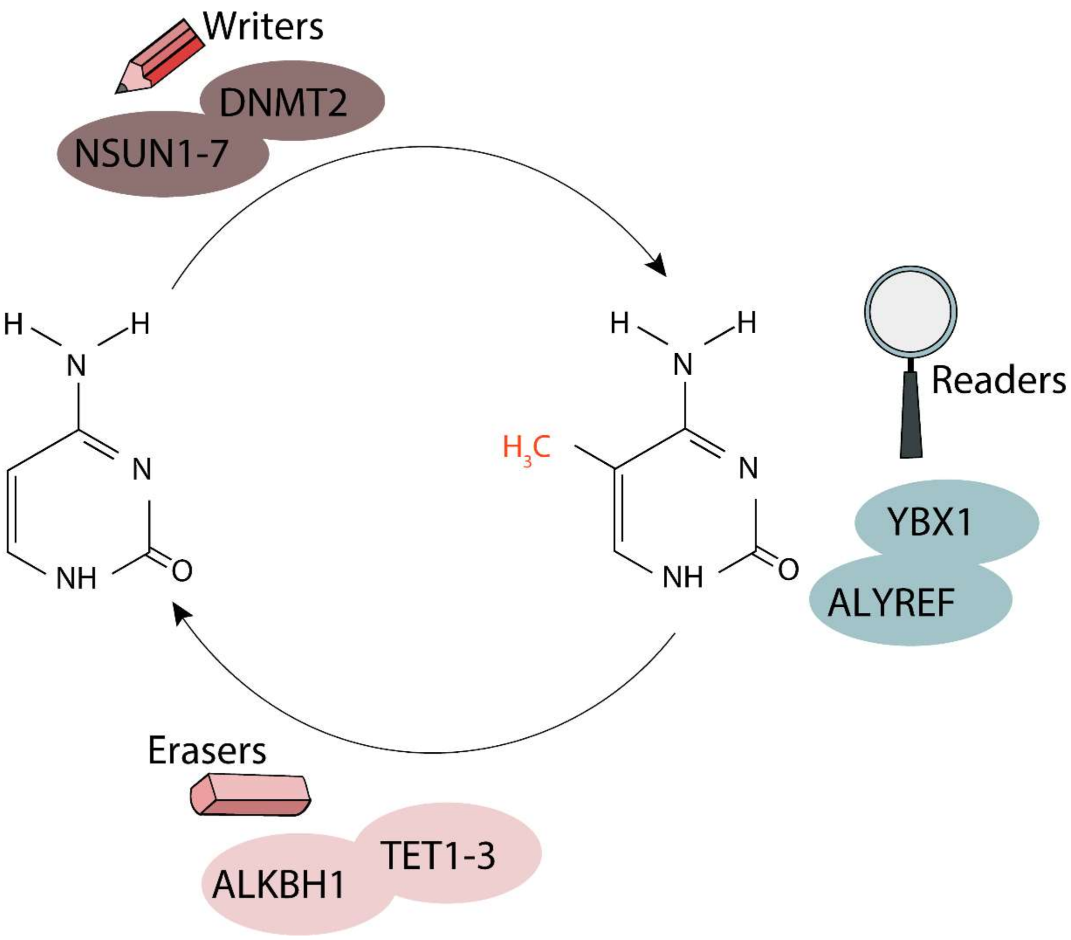

Figure S1. Overview of cellular modification of cytosine to m^{5}C in RNA (A) NSUN1-NSUN7 and DNMT2 as "writers" that catalyzes S-adenosyl methionine (SAM)-dependent methylation of the C5 of cytosine in cellular RNA. Tet1-3 ("erasers") remove the methyl group of m^{5}C; "Readers" recognize the m^{5}C mark on RNA and direct it to various outcomes;

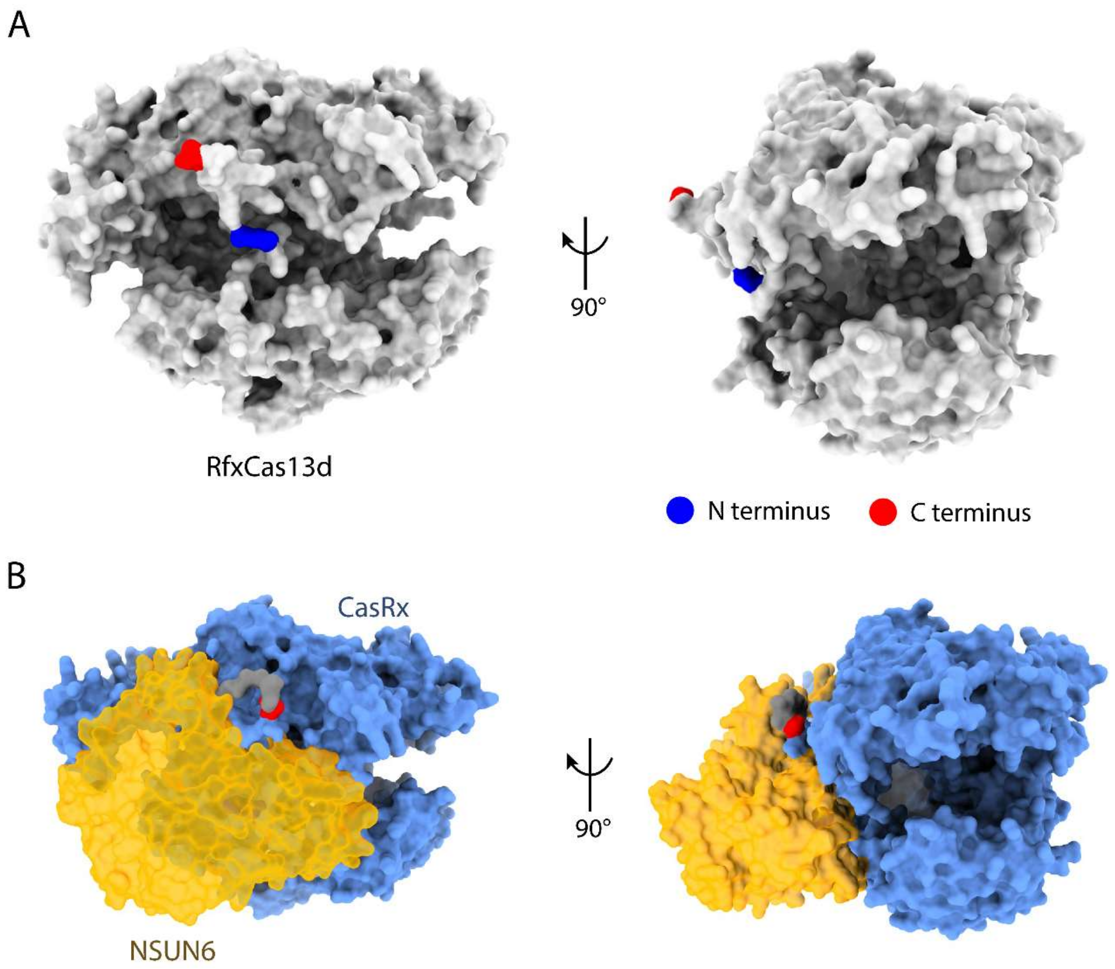

Figure S2. The predicted structure of CasRx and dCasRx-NSUN6 construction (A) The predicted structure of CasRx and the N terminus (blue) and C terminus (red) are shown. The structure Figure is generated using Alpha-Fold2; (B) The predicted structure of dCasRx-NSUN6 is shown and NSUN6 (Yellow) is fused in the C terminus (red). The linker is shown in grey.

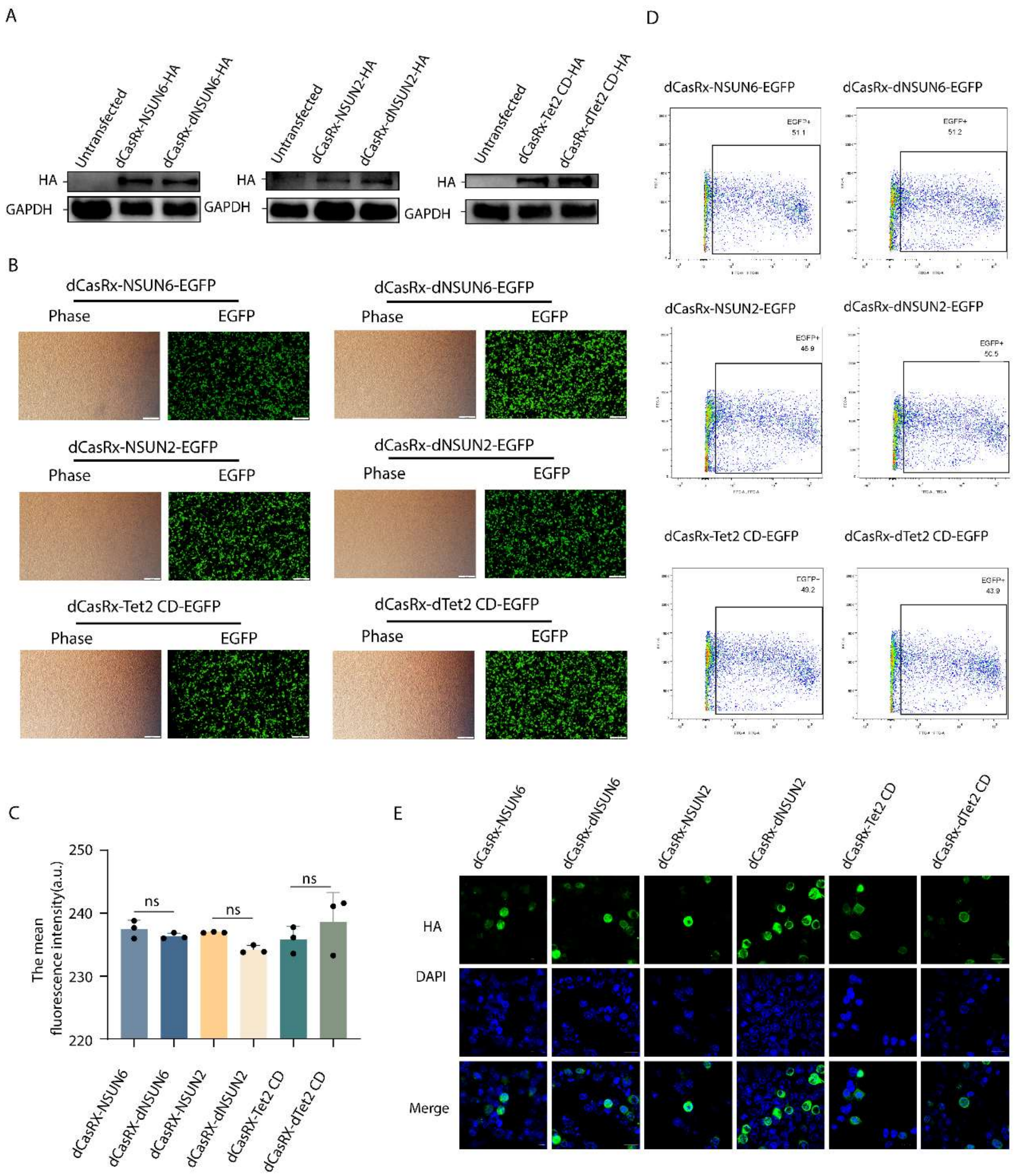

Figure S3. Expression of RCMS editors in HEK293T cells (A) Western blot results of HA-tag demonstrated expression of dCasRx epitranscriptomic editors in HEK293T cells when treated with dCasRx-NSUN6 and dCasRx-dNSUN6, dCasRx-NSUN2 and dCasRx-dNSUN2, and dCasRx-Tet2 CD and dCasRx-dTet2 CD; (B) Examination of the EGFP-positive cells by fluorescence microscopy. Scale bars, 200μm; (C) Quantification of Fluorescence intensity of EGFP according to B; error bars indicate the mean±SD. *P < 0.05, **P < 0.01, ***P < 0.001, n.s. stands for not significant by two-tailed unpaired two-sample t test (*n* = 3); (D) Quantification of the EGFP-positive cells by flow cytometry; (E) Representative immunofluorescence images of HEK293T cells transfected with HA-tagged dCasRx epitranscriptomic editors. Scale bars, 20μm.

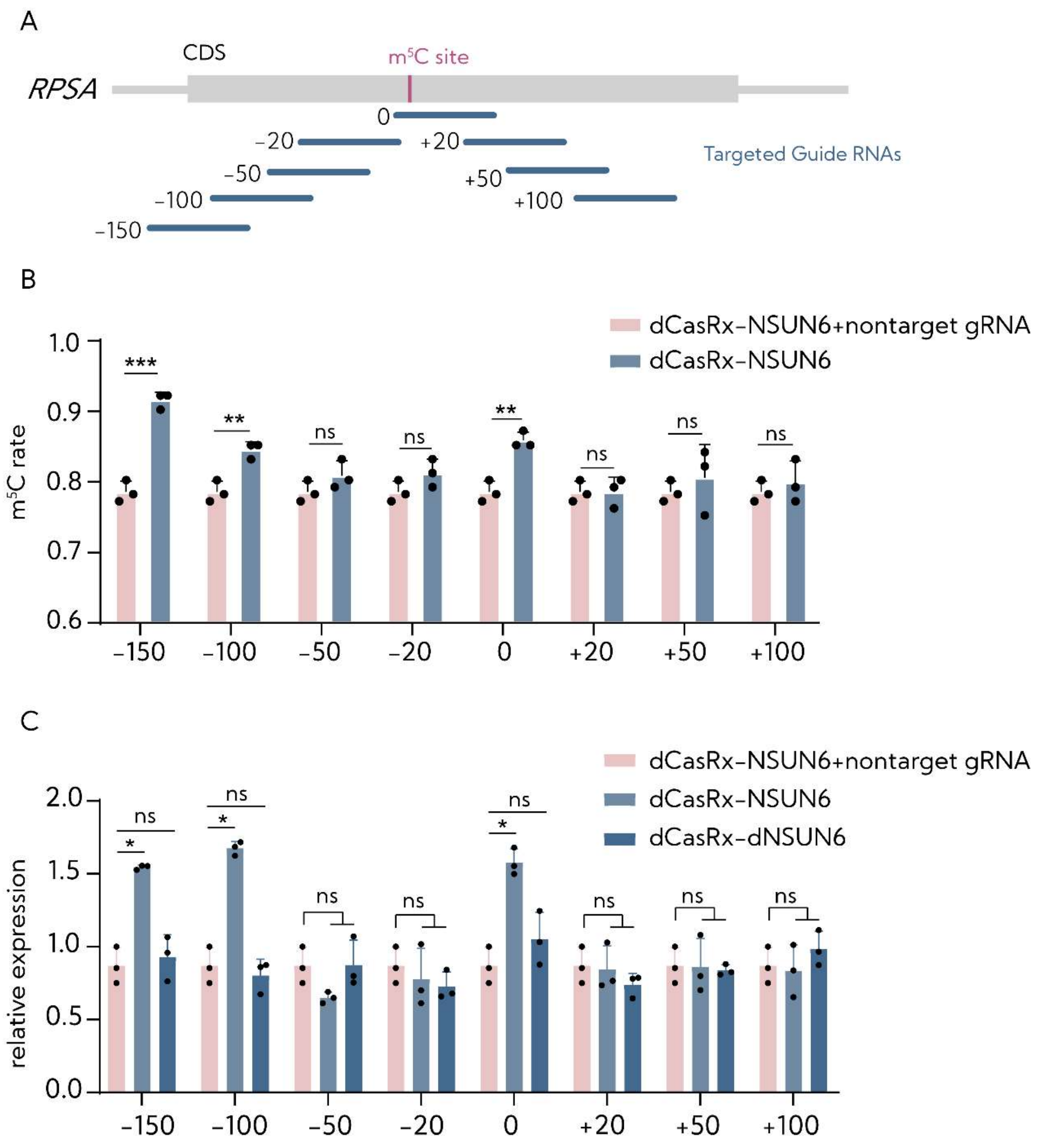

Figure S4. The RCMS methylation editors editing window on *RPSA* mRNA. (A) Schematic diagram of gRNAs designed for dCasRx-NSUN6 editor around the *RPSA* m^5C site. Each 30-nt gRNAs (dark blue) ending (-20, -50, -100, -150) or starting (+20, +50, +100) at the indicated bp from the targeted site, and 0-nt gRNA represented the gRNA covered on targeted site; (B) The m^5C ratio in m^5C234 site of *RPSA* targeted m^5C methylation by RCMS dCasRx-NSUN6 editor using sanger sequence (n = 3); (C) The mRNA levels of *RPSA* in HEK293T cells with dCasRx-NSUN6 and different gRNAs as shown in A (n = 3). Nontarget gRNA was used for nontargeting. Error bars indicate the mean ± SD. *P < 0.05, **P < 0.01, ***P < 0.001 and n.s. stands for not significant by Two-way analysis of variance (ANOVA).

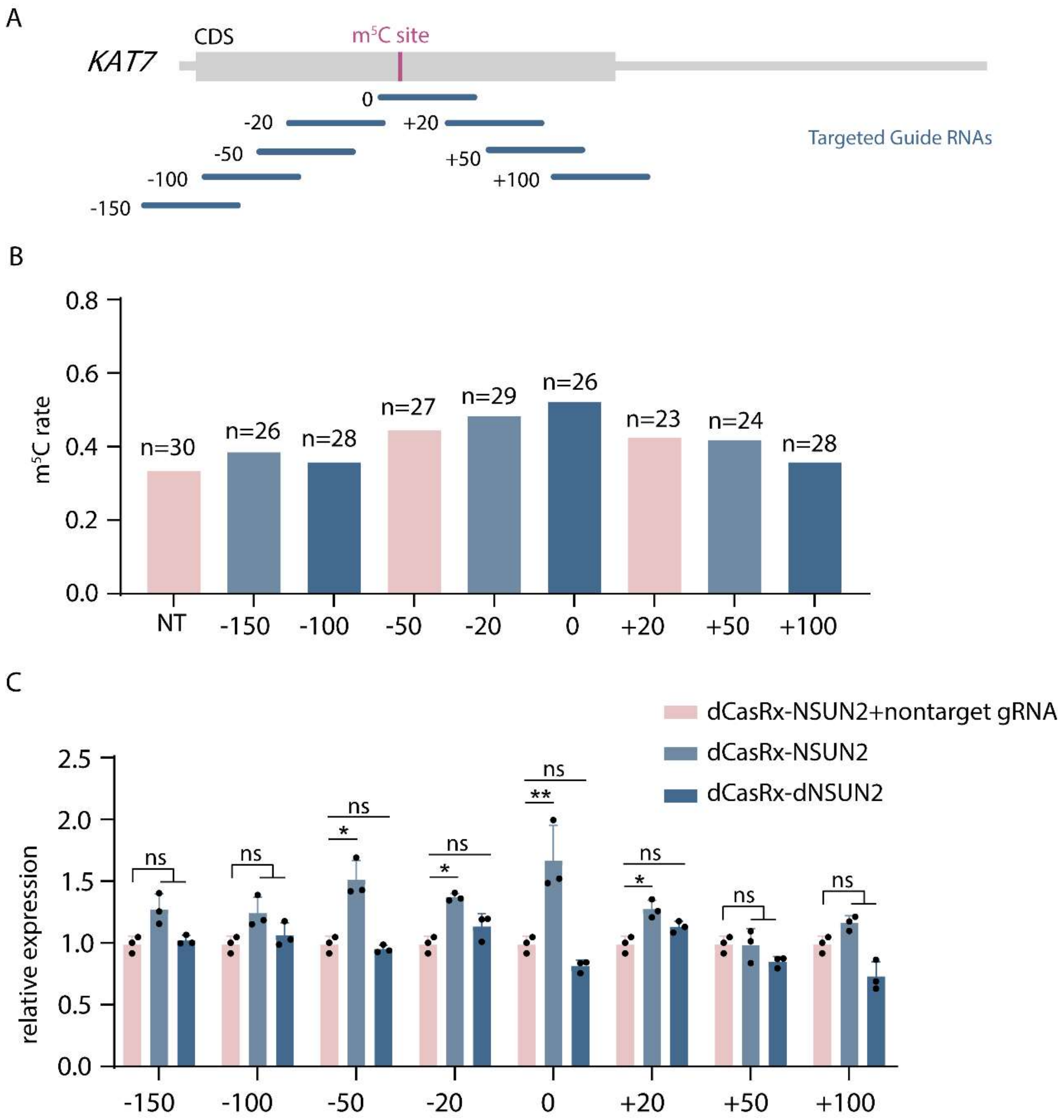

Figure S5. The RCMS methylation editors editing window on *KAT7* mRNA. (A) Schematic diagram of gRNAs designed for dCasRx-NSUN2 editor around the *KAT7* m^{5}C site. The location of gRNAs was designed as S4A; (B) The m^{5}C ratio in m^{5}C site of *KAT7* targeted by RCMS dCasRx-NSUN2 editor and the tested numbers are shown above each bar (NT (10/30), -150(10/26), -100(10/28), -50(12/27), -20(14/29), 0(12/23), +20(11/26), +50(10/24), +100(10/28); n1/n2, n1 symbolizes the number of cytosine and n2 symbolizes the total tested numbers); (C) The mRNA levels of *KAT7* in HEK293T cells transfected with dCasRx-NSUN2 and different gRNAs as shown in A (n = 3). Nontarget gRNA was used for nontargeting. Error bars indicate the mean ± SD. *P < 0.05, **P < 0.01, ***P < 0.001 and n.s. stands for not significant by two-tailed unpaired two-sample t-test.

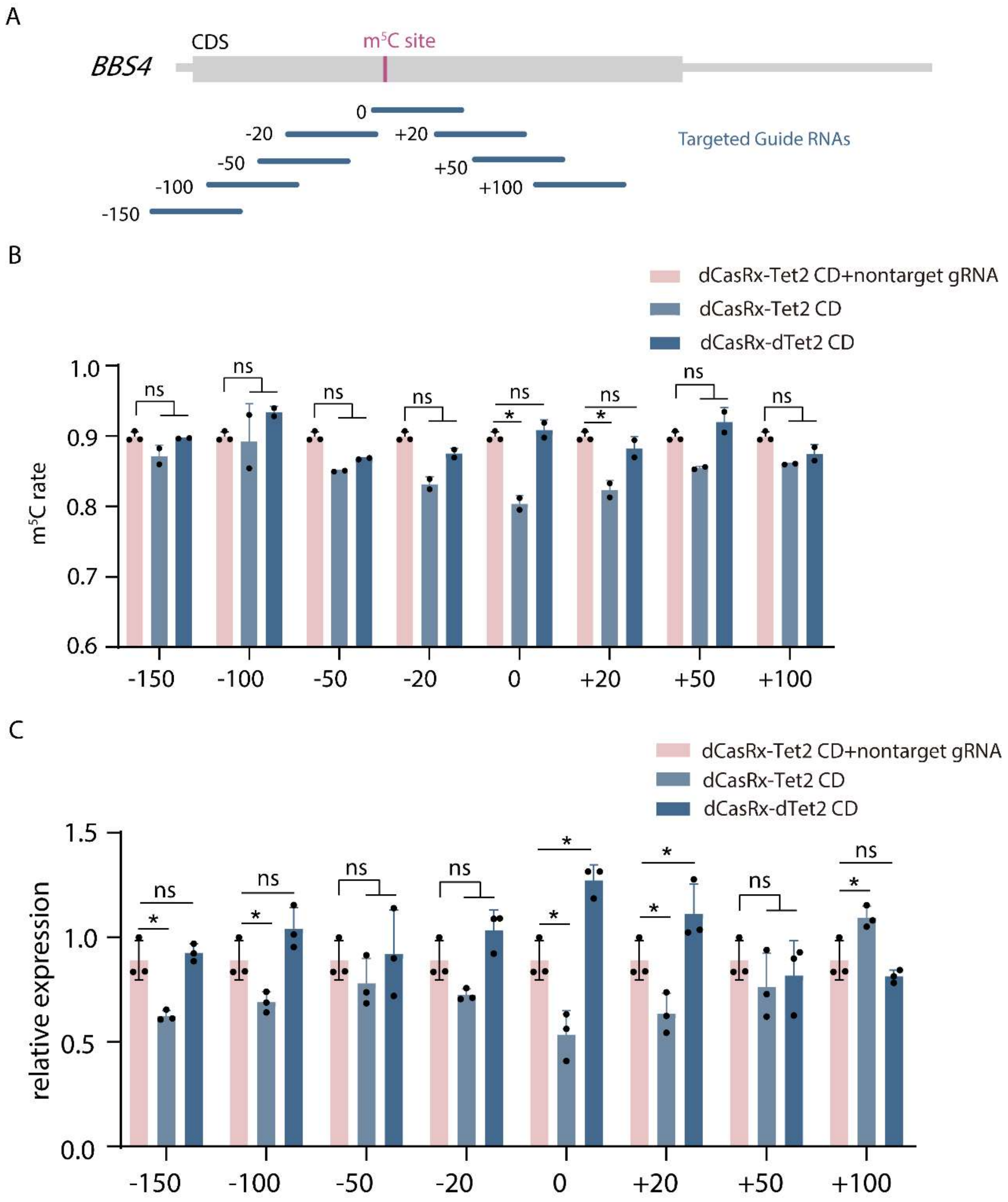

Figure S6. The RCMS demethylation editors editing window on *BBS4* mRNA. (A) Schematic diagram of gRNAs designed for dCasRx-Tet2 CD editor around the *BBS4* m^{5}C site. Each 30-nt gRNAs (purple) ending (-20, -50, -100, -150) or starting (+20, +50, +100) at the indicated bp from the targeted site, and 0-nt gRNA represented the gRNA covered on targeted site; (B) The m^{5}C ratio in m^{5}C site of *BBS4* after targeted by RCMS dCasRx-Tet2 CD editor using deep sequence HiTOM analysis (n = 3); (C) The mRNA levels of *BBS4* in HEK293T cells transfected with dCasRx- Tet2 CD and different gRNAs as shown in A (n = 3). Nontarget gRNA was used for nontargeting. Error bars indicate the mean ± SD. *P < 0.05, **P < 0.01, ***P < 0.001 and n.s. stands for not significant by Two-way analysis of variance (ANOVA) followed by Dunnett's multiple comparisons.

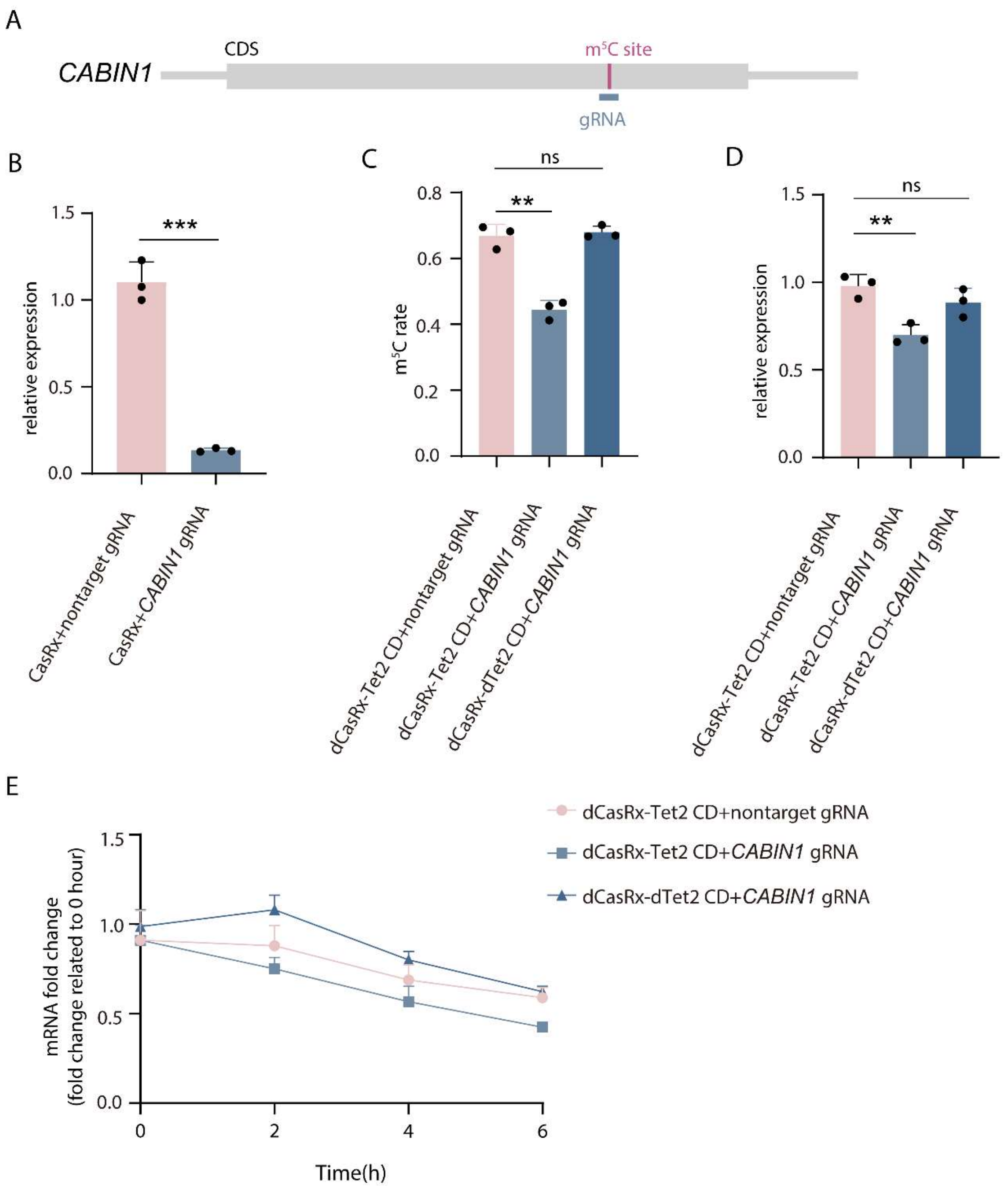

Figure S7. The targeted demethylation in the m^{5}C site of *CABIN1* transcript by the RCMS eraser dCasRx-Tet2CD editor. (A) Schematic representation of position of m^{5}C4968 site within *CABIN1* mRNA and region targeted by the gRNA; (B) Measurement of the targeting efficiency of *CABIN1* gRNA using CasRx by RT-qPCR (n = 3); (C) The m^{5}C ratio in m^{5}C4968 site of *CABIN1* targeted by dCasRx-Tet2 CD editor using deep sequence HiTOM analysis (n = 3); (D) The mRNA level of *CABIN1* in HEK293T cells with dCasRx-Tet2 CD combined with *CABIN1* gRNA or nontarget gRNA (n = 3); (E) Examination of *CABIN1* mRNA level in HEK293T cells treated with 10 mg/mL actinomycin-D, a potent transcription inhibitor, at the indicated time points by RT-qPCR (n = 3). Nontarget gRNA was used for nontargeting. Error bars indicate the mean ± SD. *P < 0.05, **P < 0.01, ***P < 0.001 and n.s. stands for not significant by two-tailed unpaired two-sample t-test.
